# Frequency-dependent fitness effects are ubiquitous

**DOI:** 10.1101/2025.08.18.670924

**Published:** 2025-08-21

**Authors:** Joao A Ascensao, Keon D Abedi, Aditya N Prasad, Oskar Hallatschek

**Affiliations:** 1 Department of Bioengineering, University of California Berkeley, CA, USA; 2 Present affiliation: Department of Organismic and Evolutionary Biology, Harvard University, MA, USA; 3 Department of Physics, University of California Berkeley, CA, USA; 4 Department of Integrative Biology, University of California Berkeley, CA, USA; 5 Peter Debye Institute for Soft Matter Physics, Leipzig University, Germany

## Abstract

In simple microbial populations, the fitness effects of most selected mutations are generally taken to be constant, independent of genotype frequency. This assumption underpins predictions about evolutionary dynamics, epistatic interactions, and the maintenance of genetic diversity in populations. Here, we systematically test this assumption using beneficial mutations from early generations of the *Escherichia coli* Long-Term Evolution Experiment (LTEE). Using flow cytometry-based competition assays, we find that frequency-dependent fitness effects are the norm rather than the exception, occurring in approximately 80% of strain pairs tested. Most competitions exhibit negative frequency-dependence, where fitness advantages decline as mutant frequency increases. Furthermore, we demonstrate that the strength of frequency-dependence is predictable from invasion fitness measurements, with invasion fitness explaining approximately half of the biological variation in frequency-dependent slopes. Additionally, we observe violations of fitness transitivity in several strain combinations, indicating that competitive relationships cannot always be predicted from fitness relative to a single reference strain alone. Through high-resolution measurements of within-growth cycle dynamics, we show that simple resource competition explains a substantial portion of the frequency-dependence: when faster-growing genotypes dominate populations, they deplete shared resources more rapidly, reducing the time available for fitness differences to accumulate. Our results demonstrate that even in a simple model system designed to minimize ecological complexity, subtle ecological interactions between closely related genotypes create frequency-dependent selection that can fundamentally alter evolutionary dynamics.

## Introduction

The fitness effects of most mutations in ecologically unstructured, well-mixed microbial populations are generally assumed to be constant, regardless of genotype frequency^1^. This assumption, rooted in classical population genetic theory^2–4^, underlies fundamental concepts from the distribution of fitness effects^5–10^ to predictions about fixation dynamics and the evolution of genetic diversity^11–14^. In microbial evolution experiments, fitness measurements typically treat beneficial mutations as having constant effects that drive predictable selective sweeps, with evolutionary outcomes determined heavily by the rank order of fitness advantages^15–17^.

However, this framework ignores a critical ecological reality: organisms do not evolve in isolation but interact with competitors, either directly (e.g. predation) or indirectly (e.g. modifying a shared environment). Consequently, selection depends on the composition of populations^18–21^. Frequency-dependent selection, where fitness effects vary with the relative abundance of genotypes, has long been recognized as a fundamental evolutionary force in natural populations which maintains genetic diversity, countering the homogenizing effects of directional selection and genetic drift^22–30^. Yet, frequency-dependent effects are typically considered exceptional rather than routine, particularly in simple laboratory environments where ecological complexity is thought to be minimal^1,31–33^.

Recent advances in microbial experimental evolution have begun to challenge this perspective. The *E. coli* Long-Term Evolution Experiment (LTEE) has revealed unexpected patterns of persistent coexistence among evolved lineages, with multiple ecotypes stably coexisting and coevolving for tens of thousands of generations^22,34–40^. These observations suggest that even in highly controlled laboratory environments, ecological interactions can generate frequency-dependent selection that fundamentally alters evolutionary dynamics^41–45^. In mixed microbial populations composed of distinct ecotypes, resource competition is expected to play a central role in the frequency-dependent selection and eco-evolutionary dynamics, since shifts in relative abundance alter the environment in ways that feed back on fitness differences^10^. The prevailing assumption, however, is that in populations composed of a single ecotype, single point mutations have negligible effects on resource usage and thus do not generate frequency-dependent fitness effects. Yet, previous work^21^ revealed that the invasion fitness of a *pykF* knockout–an important beneficial target of adaptation in the early LTEE–was significantly higher than prior measurements at high frequency^46,47^. This discrepancy suggested possible negative frequency-dependent fitness effects and prompted us to investigate how common such frequency-dependence might be among early LTEE mutations.

Here, we systematically investigate frequency-dependent fitness effects in a collection of beneficial mutations from the early generations of the *E. coli* LTEE. These mutations represent well-characterized targets of adaptation that were previously considered to have simple, unconditionally beneficial effects^47^. Using highly precise flow cytometry-based competition assays, we measure fitness effects across multiple frequencies and test fundamental assumptions about the constancy and transitivity of fitness relationships. We find that frequency-dependent fitness effects are ubiquitous, occurring in the majority of strain pairs tested. Through detailed analysis of within-growth cycle dynamics, we identify resource competition as a key mechanism underlying frequency-dependence and demonstrate that simple ecological interactions can generate complex fitness relationships, even in highly controlled laboratory environments.

The implications of widespread frequency-dependent fitness effects extend across multiple domains. If fitness effects typically depend on population composition rather than remaining constant, this would affect predictions about evolutionary dynamics, the interpretation of epistatic interactions, and the design of competition experiments to measure fitness effects. Moreover, the ubiquity of frequency-dependent effects could explain the persistence of genetic diversity in laboratory populations and provide insights into the mechanisms that generate and maintain the extraordinary diversity observed in natural microbial communities^48–52^.

## Results

### Beneficial mutations exhibit pervasive frequency-dependent fitness effects

**Figure 1.**
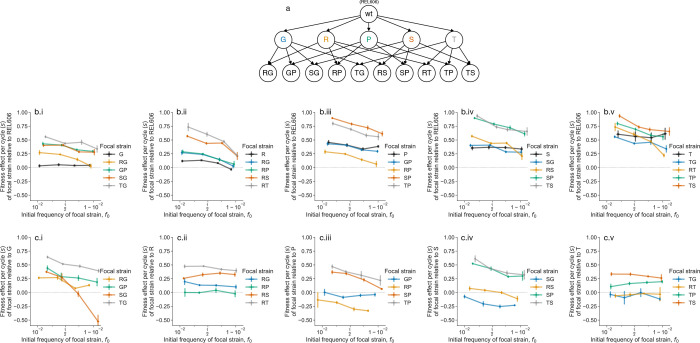
Ubiquitous frequency-dependent fitness effects. (**a**) We study a set of single and double mutants, all derived from the ancestor of the *E. coli* LTEE, REL606. Genotypes are denoted by nodes, and ancestral relationships denoted by arrows. (**b**) We measured the fitness effects, *s*, of all single and double mutants against REL606 as a function of frequency. Each subpanel (i-v) shows the measured frequency-dependent fitness effects for each single mutant and derived double mutants. Note that we plot the frequency-dependent fitness effects of double mutants twice–once on each subpanel corresponding to an immediate ancestral single mutant–to allow for comparison. (**c**) We additionally measured the frequency-dependent fitness effects of all double mutants against their single mutant ancestors. Error bars represent standard errors across biological replicates (n=4∼8) (see Table S1).

In simple, ecologically unstructured microbial populations, fitness effects of mutations are typically assumed to be constant properties, independent of their frequency in the population. To test this assumption, we measured the frequency-dependent fitness effects of a previously constructed^47^ panel of beneficial mutations from the early generations of the *E. coli* Long-Term Evolution Experiment (LTEE) (Figure 1a). These mutations represent five beneficial mutations that are common targets of adaptation in the first few thousand generations of the LTEE and were previously considered unconditionally beneficial. The mutations were reconstructed on a clean genetic background of the LTEE ancestral strain, REL606. The mutants are labeled: *G*, SNP in the *glmUS* promoter; *R*, deletion of the *rbs* operon; *P*, deletion of the *pykF* gene; *S*, SNP in the *spoT* gene; and *T*, SNP in the *topA* gene. We consider all single and double mutants.

**Figure 2.**
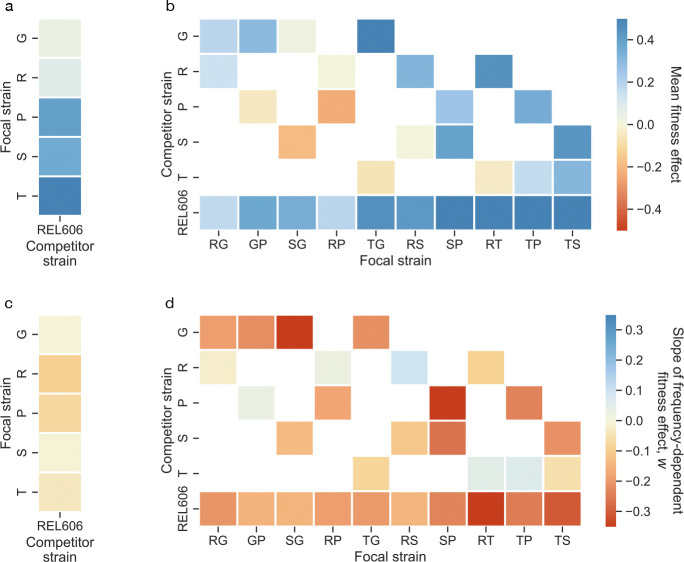
Summaries of frequency-dependent fitness effects. (**a-b**) Mean fitness effect of the focal strain relative to the competitor strain, across biological replicates and measured frequencies, of all competitions. (**c-d**) Mean slope of frequency-dependent fitness effects for all competitions, of the focal strain relative to the competitor strain. Slopes obtained through orthogonal distance regression (SI section S2.4).

To measure fitness effects via flow cytometry, we tagged each mutant with either a red or yellow fluorescent protein (RFP/YFP); the fluorescent protein gene was integrated into a putatively neutral, intergenic genomic site via a Tn7 transposon system^53^. Consistent with previous work^27^, the choice of fluorescent protein had no measurable effect on fitness (Figure S3). The LTEE populations evolve in a simple daily dilution environment, where one one-hundredth of the population is transferred to fresh glucose minimal media every 24 hours. We initiated competitions in the LTEE environment at four different starting frequencies, around 1%, 20%, 80%, and 99% (approximately equally spaced on the logit axis). We measured frequencies (f) over two growth cycles, calculating the fitness effect (s) of the focal strain relative to the competitor, on a per-cycle basis, as the slope of the logit-transformed frequencies^54,55^:

(1) $$s\left(f_{0}\right)\equiv logit\left(f_{1}\right)-logit\left(f_{0}\right),$$

where $logit(x)=log\left(\frac{x}{1-x}\right)$. We use a per-cycle fitness measure instead of a per-generation fitness measure, as the per-generation measure implicitly assumes uniform doubling times and neglects contributions like longer lag times or better survival during stationary phase–potentially mischaracterizing fitness effects^56^. Contrary to prior expectations, we observed substantial frequency-dependent effects in most strain pairs (Figures 1b–c, S1). While most focal strains maintained positive mean fitness effects relative to the ancestral competitor REL606, the magnitude of these effects varied significantly with frequency. In at least two cases (SG vs. G and RS vs. S), the frequency-dependent fitness relationship crossed zero, indicating potential for stable coexistence (Figure 1c.i&.iv). At least three double mutants (RP, SG, TG) showed lower mean fitness than their single mutant ancestors in some conditions–a signature of sign epistasis, consistent with prior data^47^ (Figure 2a–b).

Approximately 75% of competitions (at p<0.01, FDR-corrected) exhibited clear negative frequency-dependence, where fitness effects declined as mutant frequency increased (Figure 2c–d). This pattern would cause fixation dynamics to slow as beneficial mutations approach fixation, extending the time required for mutants to sweep through populations. Indeed, Wright-Fisher-like simulations (in the strong selection-weak mutation regime) reveal that the negative frequency-dependent fitness effects observed here typically increase the average mutant fixation time by hundreds of generations (Figure S6). Interestingly, two competitions (RS vs. R and TP vs. T) showed significant positive frequency-dependence. Double mutants exhibited frequency-dependent effects more frequently than single mutants–only three of five single mutants showing measurable frequency-dependence against REL606, compared to 27/30 competitions involving double mutants. The ubiquity of frequency-dependent effects across these well-characterized beneficial mutations reveals that ecological interactions are the norm rather than the exception, even in laboratory environments designed to minimize complexity.

### Predictable patterns emerge in frequency-dependent fitness effects

**Figure 3.**
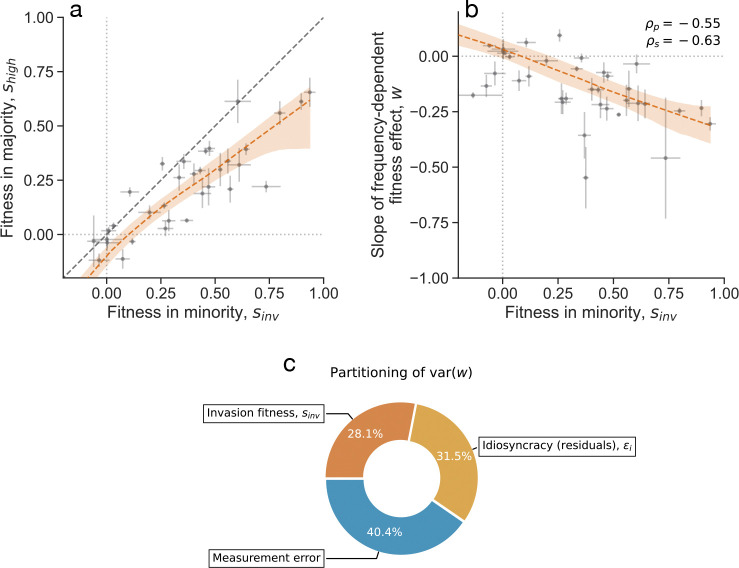
Statistical patterns of frequency-dependent fitness effects. (**a**) Comparison of low-frequency ($s_{inv}$) with high-frequency ($s_{\mathrm{high}}$) fitness effects for all competitions. (**b**) The average slope of frequency-dependent fitness effects is negatively correlated with the fitness effect of the focal strain in the minority. $\rho_{p}$ represents Pearson’s correlation; $\rho_{s}$ represents Spearman’s correlation. The red lines represent a LOWESS fit. All error bars represent standard errors. (**c**) Partitioning the variance in frequency-dependent fitness effect slopes using ANCOVA into three components: invasion fitness, biological idiosyncrasy, and measurement error.

Given the widespread frequency-dependent fitness effects among the tested clonal strains, we sought to understand if there are any predictable factors that affect the magnitude of frequency-dependence. Consistent with widespread negative frequency-dependence, fitness effects where the focal strain is in the minority, $s_{inv}$, are higher than when the focal strain is in the majority, $s_{high}$, on average (Figure 3a). Moreover, the relationship between $s_{inv}$ and $s_{high}$ shows a slight curvature at higher absolute invasion fitness effects. This curvature is reflective of a general negative dependence of the slope (w) of the frequency-dependence on $s_{inv}$ (Figure 3b). This negative correlation is quite strong, with absolute correlation coefficients around 60–70%.

We fit ANCOVA models (Supplement section S2.5) to the data to partition the variance between competitions of the frequency-dependent fitness effect slope into three components: invasion fitness, biological idiosyncracy, and measurement error. We find that measurement error can explain about 40% of the variance in w, and biological idiosyncracy (unexplained variance) and invasion fitness each explain another 30%. Thus, we are able to explain roughly half of the non-technical variation in w through invasion fitness measurements alone. The strong predictive relationship between invasion fitness and frequency-dependent slope suggests that these ecological interactions follow systematic, predictable rules.

### Fitness relationships violate transitivity assumptions

Evolutionary theory typically assumes that fitness effects are transitive and additive. Under this assumption, if we measure fitness effects $s_{ik}$ of strain i relative to strain k and $s_{jk}$ of strain j relative to strain k, we should be able to predict the fitness effect of strain i relative to strain $j,\;s_{ij}=s_{ik}-s_{jk}$. Deviations from this relationship indicate that ecological interactions affect fitness in context-dependent ways. We refer to all such deviations as fitness non-transitivity, $v=s_{ij}-s_{ik}+s_{jk}$ (observed - expected fitness effect).

**Figure 4.**
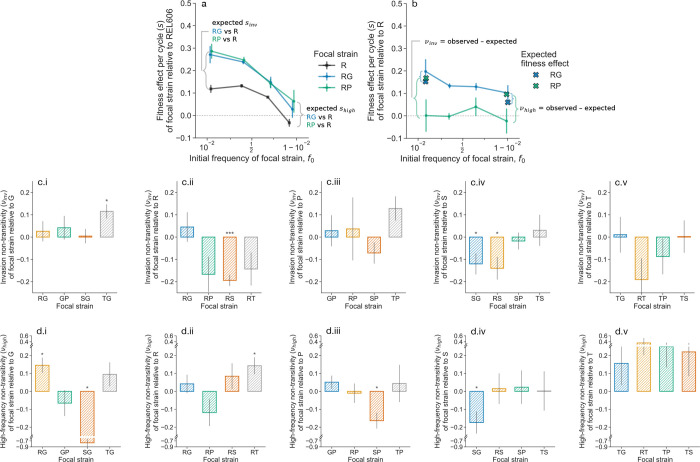
Non-transitivity in fitness effects. (**a-b**) Non-transitivity is defined as the deviation between expected fitness effects (if fitness effects between two competitions are additive) and observed fitness effects in a third experiment. Here, we show results from two definitions of non-transitivity at (**c**) low and (**d**) high frequencies; additional definitions are shown in Figure S4. Each column (i-v) represents non-transitivity of double mutants against different single mutants: (i) G, (ii) R, (iii) P, (iv) S, (v) T. Note that y-axis scales differ between rows. *p < 0.05, **p < 0.01, ***p < 0.001, post-FDR correction.

We tested transitivity by comparing predicted and observed fitness effects across strain triads, focusing on cases where one strain comprised the vast majority or minority of the population. We examined eight possible definitions of frequency-dependent non-transitivity (see Supplement section S2.2), presenting two natural cases here (Figure 4a–b; remaining cases in Figure S4).

We observed clear and significant violations of transitivity in several strain combinations, where observed fitness non-transitivity, ν, typically ranged around ±20%. Under four other definitions of non-transitivity, there are a higher number of significant cases of non-transitivity (Figure S4). These non-transitive effects indicate that fitness effects are context-dependent; they could substantially alter evolutionary dynamics by favoring or disfavoring mutations based on the composition of competing genotypes in the population.

### Niche construction drives frequency-dependent fitness effects

**Figure 5.**
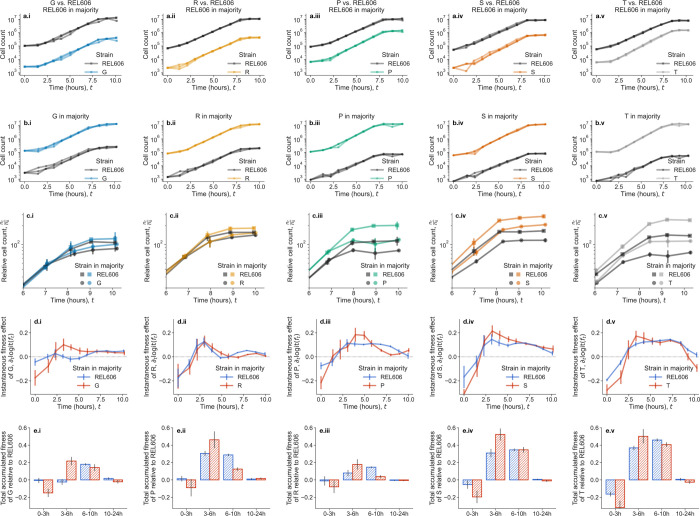
Within-growth cycle dynamics reveal ecological dynamics. (a-b) Dynamics of cell counts over the course of the first ~10 hours of the growth cycle; where either (a) REL606 is in the majority of the population, or (b) the mutant is in the majority of the population. Trajectories are shown for all three biological replicates per condition. (c) Comparison of mean relative cell count trajectories (normalized to initial count) at the end of exponential phase. Generally, when the mutant is in the majority, all strains tend to exit exponential phase earlier than when REL606 is in the majority. (d) Estimates of the instantaneous, time-dependent fitness effects of the mutant, relative to REL606. (e) Total fitness accumulated over specified intervals of time, i.e. effectively integrating the curves in (e) over intervals in time. All error bars correspond to standard errors.

To understand the ecological basis of frequency-dependent fitness effects, we measured within-growth cycle dynamics for competitions between each single mutant and REL606 at both high and low frequencies (Figure 5a–b). We subsampled cocultures approximately hourly for ten hours, covering exponential phase and the transition to stationary phase, with an additional measurement at 24 hours; population sizes are approximately constant from 10–24 hours, as expected (Figure S8).

The “instantaneous” fitness effects changed dramatically over time for each competition (Figure 5d). Even mutants (G, S, T) that showed no significant overall frequency-dependence exhibited transient frequency-dependent advantages and disadvantages that quantitatively canceled out over the full growth cycle (Figure S10). Competitions generally showed three qualitatively consistent phases (Figure 5e): (1) mutants initially grew slower than REL606, but performed relatively better when REL606 was in the majority; (2) mutants gained fitness advantages around 3–6 hours, with stronger advantages when the mutant was in the majority; and (3) around 6–10 hours, mutants generally performed better with REL606 in the majority. These frequency-dependent changes in fitness trajectories suggest that ancestral and mutant strains modify their shared environment in different ways; these environmental modifications appear strong enough to noticeably influence fitness trajectories.

We can further investigate the causes of transient fitness dynamics by examining population growth rates over time. Fitness effects and growth rates are related in a straightforward way: the difference in growth rates between mutant and ancestor equals the fitness effect, $\partial_{t}\;logit(f)=\partial_{t}\;log\left(n_{mut}\right)-\partial_{t}\;log\left(n_{wt}\right)$. Growth rates for all strains follow an inverted-U trajectory across all conditions (Figure S9). This pattern is expected for bacterial growth dynamics–growth rates typically peak in mid-exponential phase before declining toward stationary phase. However, we observed substantial quantitative differences in growth trajectories between frequency conditions. Both REL606 and mutant growth rates depend on population composition, with both strain types achieving higher growth rates when REL606 dominates, particularly at the beginning and end of exponential phase.

We believe that the late-exponential growth advantages observed when REL606 is in the majority have a simple explanation that helps account for the ubiquity of frequency-dependent fitness effects. Examining the raw growth curves (Figure 5c) reveals that most populations enter stationary phase earlier when mutants dominate. For example, when P competes with REL606 and REL606 is in the majority (Figure 5c.iii), both subpopulations remain in exponential growth until approximately t=8 hours. However, when P dominates, populations begin slowing around t=7 hours. We reasoned that this earlier end to exponential phase could reduce beneficial mutants’ ability to accumulate fitness advantages by shortening the period during which their growth advantage can be realized.

To investigate this, we analyzed a simple toy model of growth dynamics. Suppose that we are tracking the dynamics of the cell densities of a wild-type and mutant strain in a shared environment, $n_{wt}(t)$ and $n_{mut}(t)$ respectively. These strains can each grow by consuming a single, exhaustible resource R(t) initially present in the environment (e.g. glucose) at a concentration of $R_{0}$. Using a consumer-resource modeling framework, the dynamics of all three variables are,

(2) $${\dot{n}}_{wt}(t)=r_{wt}n_{wt}b(R),$$

(3) $${\dot{n}}_{mut}(t)=r_{mut}n_{mut}b(R),$$

(4) $$\dot{R}(t)=-r_{wt}a_{wt}n_{wt}b(R)-r_{mut}a_{mut}n_{mut}b(R).$$

Here, b(R(t)) captures the dependence of resource uptake on resource concentration, $r_{wt},\;r_{\mathrm{mut}}$ represent growth rates, and $a_{wt},\;a_{mut}$ represent resource conversion factors. Here, we choose b(R) to be a simple step function θ(R). The dynamics are also exactly solvable for other choices of b(R) (e.g. $b(R)=R^{n}$ or $b(R)=\frac{R}{k+R}$), and the results shown here still hold for other monotonically increasing choices of b(R) (Supplement Section S4, Figures S11–S12). We are able to analyze the dynamics analytically in two limits, in the high frequency limit ($n_{wt}(t)\gg n_{mut}(t)$) and low frequency limit $\left(n_{wt}(t)\ll n_{mut}(t)\right)$. We can show that when the wild-type dominates, resource depletion time is $T=\frac{1}{r_{wt}}log(\frac{R_{0}}{a_{wt}n_{wt}(0)}+1)$; analogously, when the mutant dominates, $T=\frac{1}{r_{mut}}log(\frac{R_{0}}{a_{mut}n_{mut}(0)}+1)$. The dependence $T\propto r_{\mathrm{major}}^{-1}$ is independent of the choice of b(R) (Supplement section S4). The fitness effect over the entire cycle is $s=T\left(r_{mut}-r_{wt}\right)$, which depends on depletion time T. And T depends inversely on the growth rate of the strain in the majority; thus, when a faster growing strain is in the majority, the resource runs out faster and fitness differences are less pronounced. Additionally, we find that non-transitive fitness effects naturally arise in our batch resource competition model (Supplement section S4). This model also predicts a negative relationship between invasion fitness and frequency-dependent slope (Figure S13), consistent with experimental observation (Figure 3b). Furthermore, our model is consistent with measurements of growth curves in all 16 strains in monoculture (Figure S14).

This model explains our key observation: when faster-growing strains dominate, resources deplete more quickly, reducing the time available for fitness differences to accumulate. This simple consequence of resource depletion provides a generic mechanism for frequency-dependent fitness effects during late (6–10 hrs) exponential phase. The additional frequency-dependent effects during the early- (0–3 hrs) and mid- (3–6 hrs) exponential phase likely reflect other ecological interactions. The total frequency-dependence then likely results from a combination of the simple consequences of nutrient limitation plus additional, possibly idiosyncratic, ecological processes.

These results demonstrate that subtle ecological interactions between closely related genotypes occur even in simple, controlled, and “constant” laboratory environments. Our model suggests that we should always expect a degree of frequency-dependence in any ecological setting involving resource depletion (including batch culture experiments), unless they are counteracted by other frequency-dependent effects.

## Discussion

Our demonstration that frequency-dependent fitness effects are ubiquitous among beneficial mutations from the early LTEE fundamentally challenges assumptions about the typicality of constant fitness effects in microbial evolution^1,47^. We find that even in highly controlled laboratory environments, subtle ecological interactions generate complex evolutionary dynamics that most models of microbial evolution do not account for.

Perhaps the most striking aspect of our results is how common frequency-dependent fitness effects prove to be. Rather than unusual cases requiring special explanation, frequency-dependence appears to be the norm rather than the exception. This finding is particularly surprising given that our study system–the LTEE environment, with its simple glucose-limited medium and controlled conditions–was specifically designed to minimize ecological complexity^1,31^. This observation may explain why coexistence has arisen so predictably in the LTEE^38^–negative frequency-dependent fitness effects that cross zero are common enough that it may be relatively easy to establish coexistence, assuming further adaptation doesn’t wipe out the nascent ecosystem.

The prevalence of frequency-dependent effects suggests that ecological interactions emerge readily from even subtle differences between closely related genotypes. We found that simple resource competition dynamics can generate frequency-dependence: when faster-growing genotypes dominate, they deplete resources more quickly, reducing the time for fitness differences to accumulate. This creates a general mechanism where fitness differences depend on population composition. However, we also observed significant frequency-dependent effects during early and mid-exponential phase, before resource depletion occurs, indicating that other ecological interactions are important contributors. Moreover, while resource competition is prevalent, it cannot reverse the sign of fitness effects. Thus, the two cases where frequency-dependent fitness effects cross zero must be driven by other ecological interactions. Future work should investigate the distribution of flavors of ecological interactions that produce frequency-dependence and examine their prevalence across diverse systems and environments.

Ultimately, our results suggest that the apparent simplicity of laboratory evolution experiments may be deceiving. Even in the most controlled environments, evolution operates through complex ecological interactions that deform fitness landscapes, determine competitive outcomes, and influence the maintenance of genetic diversity. Rather than simple hill-climbing on static fitness landscapes, microbial communities navigate dynamic fitness spaces shaped by ecological interactions, epistatic constraints, and environmental feedback.

## Methods

See supplementary information.

## Supplementary Material

Supplement 1
