# Supplementary material for: Frequency-dependent fitness effects are ubiquitous
Source: bioRxiv. 2025 Aug 21:2025.08.18.670924. Preprint. [Version 1] doi: 10.1101/2025.08.18.670924 (PMC12393377; doi:10.1101/2025.08.18.670924)
Supplement: Supplement 1 [file NIHPP2025.08.18.670924v1-supplement-1.pdf]

bioRxiv preprint doi: https://doi.org/10.1101/2025.08.18.670924; this version posted August 21, 2025. The copyright holder for this preprint (which was not certified by peer review) is the author/funder, who has granted bioRxiv a license to display the preprint in perpetuity. It is made available under aCC-BY 4.0 International license.

Frequency-dependent fitness effects are ubiquitous

Supplementary Information

Joao A Ascensao, Keon D Abedi, Aditya N Prasad, Oskar Hallatschek

2025

Contents

S1 Experimental methods **3**

S1.1 Growth conditions, media and strains . . . 3

S1.2 Fluorescent tagging . . . 3

S1.3 Flow cytometry . . . 3

S2 Frequency-dependent fitness measurements **4**

S2.1 Quantifying fitness effects . . . 4

S2.2 Quantifying non-transitivity . . . 4

S2.3 Frequency-dependent epistasis . . . 6

S2.4 Quantifying frequency-dependent slopes and curvature . . . 6

S2.5 ANCOVA model . . . 7

S3 Within-cycle time-courses **9**

S3.1 Coculture experiments . . . 9

S3.2 Measuring growth curves via plate reader . . . 10

S4 Batch culture resource competition model **10**

bioRxiv preprint doi: https://doi.org/10.1101/2025.08.18.670924; this version posted August 21, 2025. The copyright holder for this preprint (which was not certified by peer review) is the author/funder, who has granted bioRxiv a license to display the preprint in perpetuity. It is made available under aCC-BY 4.0 International license.

List of Figures

S1 Ubiquity of frequency-dependence 13
S2 Example of flow cytometry gating strategy 14
S3 Fluorophores do not differentially impact fitness 15
S4 Non-transitivity of fitness effects 16
S5 Frequency-dependent epistasis 17
S6 Fixation time simulation estimates 17
S7 Statistical relationships between invasion/high-frequency fitness effects and frequency dependence 18
S8 Within-cycle population dynamics 18
S9 Within-cycle growth rates 19
S10 Accumulated frequency-dependence 19
S11 Simulations of resource competition dynamics 20
S12 Frequency-dependence arising from resource competition dynamics 21
S13 Frequency-dependent slopes predicted from resource competition dynamics 22
S14 Growth curves of monocultures 23

List of Tables

S1 Frequency-dependent slopes. 7
S2 Results from ANCOVA models. 9

bioRxiv preprint doi: https://doi.org/10.1101/2025.08.18.670924; this version posted August 21, 2025. The copyright holder for this preprint (which was not certified by peer review) is the author/funder, who has granted bioRxiv a license to display the preprint in perpetuity. It is made available under aCC-BY 4.0 International license.

S1 Experimental methods

S1.1 Growth conditions, media and strains

All experiments described here were conducted in Davis Minimal (DM) base medium, composed of 5.36 g/L potassium phosphate (dibasic), 2 g/L potassium phosphate (monobasic), 1 g/L ammonium sulfate, 0.5 g/L sodium citrate, 0.01% magnesium sulfate, and 0.0002% thiamine HCl. The specific medium used in both the Long-Term Evolution Experiment (LTEE) and all assays presented here was DM25–i.e. DM supplemented with 25 mg/L glucose.

For coculture experiments, we began by inoculating the desired strain into 1 mL of LB supplemented with 0.2% glucose and 20 mM pyruvate directly from glycerol stock. Following overnight incubation, cultures were washed three times in DM0 (DM lacking a carbon source) by centrifugation at $2500\times g$ for 3 minutes, removal of the supernatant, and resuspension in DM0. The washed culture was then diluted 1:1000 into 1 mL of DM25 in a glass tube. In general, we grew 1 mL cultures in glass 96-well plates (Thomas Scientific 6977B05). Cultures were incubated at 37°C for 24 hours in a shaking incubator. The following day, cultures were transferred 1:100 into fresh DM25 and incubated under identical conditions. After another 24 hours of growth, we mixed selected cultures at defined frequencies and diluted the mixtures 1:100 into fresh DM25. Cultures were incubated again for 24 hours under the same conditions, after which experimental measurements were initiated.

S1.2 Fluorescent tagging

Strains carried fluorescent protein markers integrated at the *attTn7* locus via a miniTn7 transposon system, as previously described [1, 2]. Each strain either expressed sYFP2 or mScarlet-I. Briefly, we transformed a conjugative *E. coli* donor strain (MFDpir; DAP auxotroph) with a temperature-sensitive plasmid that expresses the miniTn7 machinery, with a chloramphenicol resistance gene and either sYFP2 or mScarlet-I between right and left Tn7 recognition sequences. We then conjugated the donor with the strain of interest by growing the strains together on solid media (LB/agar supplemented with 0.2% glucose and 0.3 mM DAP) for about 24 hours at 30°C. After growth, we scraped up the resulting lawn, washed three times in DM0, and streaked the culture on DM2000/agar plates supplemented with 20 µg/mL chloramphenicol. After 48 hours of growth at 37°C, we picked several candidate transconjugant colonies, testing for successful integration via PCR, and for successful loss of the plasmid by ability to grow on LB/ampicillin media (plasmid backbone has an ampicillin resistance gene). Successful clones were grown in DM2000 and saved as glycerol stocks.

S1.3 Flow cytometry

All population-level measurements were performed using a ThermoFisher Attune Flow Cytometer (2017 model) located at the UC Berkeley QB3 Cell and Tissue Analysis Facility (CTAF). Samples were loaded into a round-bottom 96-well plate compatible with the autosampler. For each run, the instrument performed one wash and mixing cycle before measurement. To prevent cross-contamination, 50µL of bleach was run through the autosampler between samples.

Fluorescence detection was performed as follows: sYFP2 using the BL1 channel (488 nm laser, 530/30 nm bandpass filter); and mScarlet-I using the YL2 channel (561 nm laser, 620/15 nm bandpass filter). Cell counts and strain frequencies were extracted from raw data using a previously validated analysis pipeline [1]. We applied a previously described gating strategy [1], using threshold-based gates to distinguish fluorescent events from debris and background noise (see Figure S2).

bioRxiv preprint doi: https://doi.org/10.1101/2025.08.18.670924; this version posted August 21, 2025. The copyright holder for this preprint (which was not certified by peer review) is the author/funder, who has granted bioRxiv a license to display the preprint in perpetuity. It is made available under aCC-BY 4.0 International license.

S2 Frequency-dependent fitness measurements

To measure frequency-dependent fitness effects of different pairs of clonal strains, we first prepared cultures as described in section S1.1. For each pair of strains, one was tagged with YFP, and the other RFP. We mixed each pair of strains at four volumetric fractions–0.01, 0.2, 0.8, and 0.99 (approximately equally spaced in logit space). We measured the relative frequencies of each strain at the end of a growth cycle via flow cytometry (day 0), then we propagated the cultures as usual (1:100 dilution into DM25). At the end of the second growth cycle, we measured the relative frequencies once more using flow cytometry (day 1). We generally used between 4 and 6 independent biological replicates for each measurement.

S2.1 Quantifying fitness effects

From the frequency measurements of clonal strains at days 0 and 1, we sought to quantify the relative fitness effects for each experiment. We denote $f_{ijk_r}(t)$ as the measured frequency of the focal strain i, relative to strain j, for experiment k_r at time t. As previously mentioned, for any given set of experimental conditions (two strains and initial frequency), we will generally have 4-6 biological replicates; we denote k as a given experiment with a set initial frequency, and r as a given biological replicate. Then, following the standard population genetic definition of fitness effects, we calculate the fitness effect in a given experiment as,

$$s_{ijk_r} = \text{logit}(f_{ijk_r}(1)) - \text{logit}(f_{ijk_r}(0)) \ . \tag{S1}$$

We noticed that the calculated s_{ijk_r} of a few biological replicates appeared to be significantly different from the remaining replicates. It appeared that most of these instances were due to apparent errors in the flow cytometer, e.g. early termination, fluidics errors, bubbles, etc. Thus, we excluded outliers from further analyis. We defined an outlier as any s_{ijk_r} that is more than three standard deviations away from the median between all biological replicates, where standard deviation is approximated by the median absolute deviation,

$$\hat{\sigma}_{ijk} = 1.4826 \underset{r}{\text{med}} |s_{ijk_r} - \underset{r}{\text{med}}(s_{ijk_r})| \tag{S2}$$

After outlier filtering, we computed our final estimates of s_{ijk} by simply averaging over s_{ijk_r} for all biological replicates r. We obtained standard errors for s_{ijk} and the initial frequency $f_{ijk}(0)$ by using the sample standard deviation.

S2.2 Quantifying non-transitivity

Under typical null models used in evolutionary biology, fitness effects are generally assumed to be additive and transitive. This arises from an assumption that the fitness of a clone is independent of the pairwise competition used to measure fitness effects. To see this, we note that the fitness effect, $s_{ij}(E)$, in environment E, of a given clone i relative to a competitor j can generically be decomposed into the difference in *fitness*, $x(E)$, of each clone,

$$s_{ij}(E) = x_i(E) - x_j(E) \tag{S3}$$

$$= \Delta\text{logit}(f_{ij}) = \Delta \log(n_i) - \Delta \log(n_j) \ . \tag{S4}$$

We denote f_{ij} as the frequency of i relative to j, and n_i as the population size of i. The environment E might be set either by any ecological interactions, or abiotic factors. If fitnesses are constant across competitions/environments, then if we measure fitness effects of a triplet of clones, we should recover a simple relationship between the measurements,

$$s_{12} - s_{13} = x_1 - x_2 - x_1 + x_3 = s_{32} \ . \tag{S5}$$

bioRxiv preprint doi: https://doi.org/10.1101/2025.08.18.670924; this version posted August 21, 2025. The copyright holder for this preprint (which was not certified by peer review) is the author/funder, who has granted bioRxiv a license to display the preprint in perpetuity. It is made available under aCC-BY 4.0 International license.

However, in general, this relationship will not hold if fitnesses depend on the specific ecological interactions of a given competition. We can quantify the deviation from transitivity (non-transitivity) between competition triplets with a quantity ν,

$$\nu = s_{13} - s_{12} + s_{32} \ . \tag{S6}$$

Of course, under this definition, $\nu = 0$ when none of the three clones have competition-dependent fitnesses.

However, in the case that fitness effects are frequency-dependent, we must choose frequencies to use for fitness effect comparisons. We choose to focus on the cases where one strain is in the vast majority or minority of the population, i.e. when one of the frequencies tends to zero. We reasoned that this regime would allow for simplified interpretation of non-transitivity, as the environment will be dominated by the effects of the strain in the majority. Here, we denote E_i as the environment that is induced when strain i is in the vast majority of the population, $f_i \to 1$. Under this constraint, there are eight possible different definitions of frequency-dependent non-transitivity. Two of these definitions appear especially natural (which we highlight in the main text): the case in which all environments are dominated by the competitor strains, comparing solely invasion fitness effects,

$$\nu_{inv} = s_{13}(E_3) - s_{12}(E_2) + s_{32}(E_2) \tag{S7}$$

$$= x_1(E_3) - x_1(E_2) - x_3(E_3) + x_3(E_2) \ , \tag{S8}$$

and the case in which all environments are dominated by the focal strains, comparing solely high-frequency fitness effects,

$$\nu_{high} = s_{13}(E_1) - s_{12}(E_1) + s_{32}(E_3) \tag{S9}$$

$$= x_3(E_3) - x_3(E_1) - x_2(E_3) + x_2(E_1) \ . \tag{S10}$$

We see that the fitness of one strain cancels out in each definition, and we're left comparing the fitness of two different strains in two different environments. In the case of quantifying the non-transitivity in our experimental data, we assign the labels: 1=double mutant; 2=wild-type (REL606); 3=single mutant. We show the results of such quantification in Figure 2. There are four other ways to construct defintions of non-transitivity so that the fitness of one strain gets canceled out,

$$\nu^3_{inv} = s_{13}(E_3) - s_{12}(E_2) + s_{32}(E_3) \tag{S11}$$

$$= x_1(E_3) - x_1(E_2) - x_2(E_3) + x_2(E_2) \tag{S12}$$

$$\nu^2_{high} = s_{13}(E_1) - s_{12}(E_1) + s_{32}(E_2) \tag{S13}$$

$$= x_3(E_2) - x_3(E_1) - x_2(E_2) + x_2(E_1) \tag{S14}$$

$$\nu^1_{inv} = s_{13}(E_1) - s_{12}(E_2) + s_{32}(E_2) \tag{S15}$$

$$= x_1(E_1) - x_1(E_2) - x_3(E_1) + x_3(E_2) \ , \tag{S16}$$

$$\nu^3_{high} = s_{13}(E_3) - s_{12}(E_1) + s_{32}(E_3) \tag{S17}$$

$$= x_1(E_3) - x_1(E_1) - x_2(E_3) + x_2(E_1) \ . \tag{S18}$$

bioRxiv preprint doi: https://doi.org/10.1101/2025.08.18.670924; this version posted August 21, 2025. The copyright holder for this preprint (which was not certified by peer review) is the author/funder, who has granted bioRxiv a license to display the preprint in perpetuity. It is made available under aCC-BY 4.0 International license.

We show the results of quantifying non-transitivity under these definitions in Figures **xx and xx**. There are two additional ways to construct definitions of frequency-dependent non-transitivity such that none of the fitnesses get canceled out; these two definitions are simply linear combinations of prior definitions.

We sought to quantify not only the point estimates of ν, but also estimates of uncertainty. We computed the sample standard error of ν from the standard errors of the fitness effects $\sigma_\nu = \sqrt{\sigma_{s_{13}}^2 + \sigma_{s_{12}}^2 + \sigma_{s_{32}}^2}$. Then, we sought to compute p-values, testing the null hypothesis that $\nu = 0$. We used a one-tailed, one-sample t-test, where we combined the degrees of freedom from all fitness effects using the standard Welch–Satterthwaite equation,

$$\mathrm{df}_\nu = \frac{\left(\sum\limits_{i \in \{13,12,32\}} \sigma_i^2\right)^2}{\sum\limits_{i \in \{13,12,32\}} \left(\frac{\sigma_i^4}{\mathrm{df}_i}\right)} \tag{S19}$$

We used a standard Benjamini-Hochberg FDR correction to correct the p-values for multiple testing.

S2.3 Frequency-dependent epistasis

We wish to quantify additive and epistatic coefficients of double and single mutants. Our situation differs from the usual case, as we must compare fitness effects across different environments. For simplicity, we focus on fitness effects at invasion and high frequencies.

For any given environment E where a given clonal strain is in the majority, the additive coefficient of a given single mutant i, we simple call the additive coefficient the measured fitness effect $s_i(E)$. Then, to quantify the epistasis between two mutations i and j, we simply take the difference between the observed fitness effect of the double mutant from the expected value under additivity, $s_{ij}(E) - s_i(E) - s_j(E)$. Note that we drop the factor of two that appear in some models of epistasis. We calculate p-values as in the previous section, through standard one-sample t-tests. We present the results of this analysis in Figure S5.

S2.4 Quantifying frequency-dependent slopes and curvature

We wished to estimate the average slope (w_i) and curvature (y_i) of the frequency-dependent fitness effects that we measured, for each set of competitions i. Both frequencies and fitness effects have experimental noise associated with each measurement, which we wished to incorporate into our estimates of w_i and y_i. We assume gaussian errors on both the measured frequencies, $\hat{f}_{ij}$, and measured fitness effects, $\hat{s}_{ij}$, for each frequency measurement j. We took the measurement errors as the empirical standard errors, estimated from biological replicates. To estimate the slopes, we modeled the frequencies and fitness effects as,

$$\hat{f}_{ij} \sim \mathcal{N}(f_{ij}, \hat{\sigma}_{fij}^2)\ , \tag{S20}$$

$$\hat{s}_{ij} \sim \mathcal{N}(a_i + w_i f_{ij}, \hat{\sigma}_{sij}^2)\ . \tag{S21}$$

We used orthogonal distance regression to fit the slopes and intercepts, independently for each competition i, using the implementation in scipy (`scipy.odr`). Results are shown in table S1.

We also obtain an estimate of the error on the slope, $\hat{\sigma}_{w,i}$. Analogously, to estimate the curvatures y_i, we modeled the frequencies and fitness effects as,

$$\hat{f}_{ij} \sim \mathcal{N}(f_{ij}, \hat{\sigma}_{fij}^2)\ , \tag{S22}$$

$$\hat{s}_{ij} \sim \mathcal{N}(a_i + w_i f_{ij} + y_i f_{ij}^2, \hat{\sigma}_{sij}^2)\ . \tag{S23}$$

bioRxiv preprint doi: https://doi.org/10.1101/2025.08.18.670924; this version posted August 21, 2025. The copyright holder for this preprint (which was not certified by peer review) is the author/funder, who has granted bioRxiv a license to display the preprint in perpetuity. It is made available under aCC-BY 4.0 International license.

Focal strain	Competitor strain	Slope	Slope standard error	n	p-value
G	REL606	-0.0024889	0.01180581	6	0.06941898
GP	G	-0.2179709	0.06161893	4	8.08E-05
GP	P	0.03220586	0.04547284	4	0.04352671
GP	REL606	-0.1512275	0.01652409	5	0
P	REL606	-0.0733617	0.04606334	4	0.00983512
R	REL606	-0.090475	0.04590815	6	0.00562485
RG	G	-0.1912887	0.0690716	4	0.00076575
RG	R	-0.020408	0.02321081	4	0.03670344
RG	REL606	-0.2071988	0.0542102	5	2.84E-05
RP	P	-0.1759485	0.01366787	4	0
RP	R	0.02256056	0.03657605	4	0.04741398
RP	REL606	-0.1907273	0.02664407	5	3.50E-13
RS	R	0.09397102	0.02805008	4	0.00014255
RS	REL606	-0.1468253	0.08173802	5	0.00804981
RS	S	-0.1101542	0.04641053	4	0.00211459
RT	R	-0.0896636	0.02113084	4	5.51E-06
RT	REL606	-0.4591719	0.27264766	5	0.00987408
RT	T	0.04772878	0.00511884	4	0
S	REL606	-0.0078465	0.02004612	8	0.05961288
SG	G	-0.5475973	0.13854724	4	1.79E-05
SG	REL606	-0.1491037	0.05123373	5	0.00054168
SG	S	-0.1340259	0.05006652	4	0.00096906
SP	P	-0.3565837	0.10492876	4	0.00012712
SP	REL606	-0.2338673	0.03654203	5	5.83E-11
SP	S	-0.2631948	0.0078109	4	0
T	REL606	-0.034554	0.04241604	8	0.03893214
TG	G	-0.2152041	0.06508236	4	0.00015737
TG	REL606	-0.1989299	0.07064311	5	0.00069469
TG	T	-0.0775731	0.05392908	4	0.01503126
TP	P	-0.2362316	0.05406085	4	3.73E-06
TP	REL606	-0.2464382	0.01413841	5	0
TP	T	0.06200534	0.02103895	4	0.00050637
TS	REL606	-0.304736	0.03012838	5	0
TS	S	-0.2121762	0.08025992	4	0.00102533
TS	T	-0.0565843	0.01190019	4	6.62E-07

Table S1: **Frequency-dependent slopes for each competition.** n is the number of biological replicates for a given competition. p-values are computed with a null hypothesis that the slopes are zero, and are corrected with a standard Benjamini-Hochberg FDR correction.

S2.5 ANCOVA model

Here we develop an ANCOVA model to model the slope of frequency-dependent fitness effects, w_i. We consider three models: (1) a model that only considers measurement error and biological idiosyncrasy (i.e. unexplained variance/residuals), (2) a model that adds the invasion fitness, $s_{inv,i}$, as a covariate, and (3) a model that adds the high-frequency fitness, $s_{high,i}$, as a covariate. We only consider competitions where the focal strain has a higher number of mutations than the competitor strain.

For all of the models detailed below, we found the associated likelihoods to compute a maximum likelihood estimate of all parameters. We summarize the results from all of the models in Table S2.

bioRxiv preprint doi: https://doi.org/10.1101/2025.08.18.670924; this version posted August 21, 2025. The copyright holder for this preprint (which was not certified by peer review) is the author/funder, who has granted bioRxiv a license to display the preprint in perpetuity. It is made available under aCC-BY 4.0 International license.

Model 1 We begin by modeling the slope of frequency-dependent fitness effects for each pairwise competition i, w_i, as linear combination of three components,

$$w_i = a + c_i + \epsilon_i \ , \tag{S24}$$

$$c_i \sim \mathcal{N}(0, \hat{\sigma}^2_{w,i}) \ , \tag{S25}$$

$$\epsilon_i \sim \mathcal{N}(0, \sigma^2_d) \ . \tag{S26}$$

Here, c_i the measurement error, ϵ_i is unexplained variance, and a is the offset. We model c_i as a normal random variable with a known standard deviation, as the sample sizes used to estimate w_i are generally large enough such that the errors should be approximately normal. The likelihood for this model, combining all measurements, is

$$p(\{w_i\}|\sigma^2_d, \{\hat{\sigma}^2_{w,i}\}) = \prod_i p(w_i|\sigma^2_d, \hat{\sigma}^2_{w,i}) \tag{S27}$$

$$= \prod_i \int_{-\infty}^{\infty} d\epsilon_i \, p(w_i|\hat{\sigma}^2_{w,i}, \epsilon_i) p(\epsilon_i|\sigma^2_d) \tag{S28}$$

$$= \prod_i f_N(w_i; a, \sigma^2_d + \hat{\sigma}^2_{w,i}) \ . \tag{S29}$$

Here we denote $f_N(x; \mu, \sigma^2)$ as a normal distribution of a random variable x with mean μ and variance σ^2.

Model 2 We now extend our model of w_i to include the invasion fitness, $s_{inv,i}$, as a covariate,

$$w_i = a + b_{inv} s_{inv,i} + c_i + \epsilon_i \ , \tag{S30}$$

$$s_{inv,i} \sim t(\hat{s}_{inv,i}, \hat{\sigma}^2_{inv,i}, \mathrm{df}_i) \ . \tag{S31}$$

Here, $\hat{s}_{inv,i}$ is the sample mean invasion fitness of competition i, $\hat{\sigma}^2_{inv,i}$ is the sample variance of the invasion fitness, and $\mathrm{df}_i = n_i - 1$ are the degrees of freedom, related to the number of biological replicates for that measurement. We model $s_{inv,i}$ as a non-centered, scaled t-distributed random variable, as we assume that underlying measurement error distribution is gaussian, but n_i is typically small, so we must account for errors in the estimated sample variance. Specifically, we use a probability distribution function of the form,

$$p(s_{inv,i}|\hat{s}_{inv,i}, \hat{\sigma}^2_{inv,i}, \mathrm{df}_i) = \frac{\Gamma\left(\frac{\mathrm{df}_i+1}{2}\right)}{\sqrt{\pi \mathrm{df}_i}\Gamma\left(\frac{\mathrm{df}_i}{2}\right)\hat{\sigma}_{inv,i}} \left(1 + \frac{(s_{inv,i} - \hat{s}_{inv,i})^2}{\hat{\sigma}^2_{inv,i}\mathrm{df}_i}\right)^{-(\mathrm{df}_i+1)/2} . \tag{S32}$$

The full likelihood of the model is then

$$p(\{w_i\}|b_{inv}, \sigma^2_d, \{\hat{\sigma}^2_{w,i}\}, \hat{s}_{inv,i}, \hat{\sigma}^2_{inv,i}, \mathrm{df}_i) \tag{S33}$$

$$= \prod_i \int_{-\infty}^{\infty} d\epsilon_i \int_{-\infty}^{\infty} ds_{inv,i} \, p(w_i|\hat{\sigma}^2_{w,i}, \epsilon_i, s_{inv,i}) p(\epsilon_i|\sigma^2_d) p(s_{inv,i}|\hat{s}_{inv,i}, \hat{\sigma}^2_{inv,i}, \mathrm{df}_i) \tag{S34}$$

$$= \prod_i \int_{-\infty}^{\infty} ds_{inv,i} \, f_N(w_i; a, \sigma^2_d + \hat{\sigma}^2_{w,i}) p(s_{inv,i}|\hat{s}_{inv,i}, \hat{\sigma}^2_{inv,i}, \mathrm{df}_i) \ . \tag{S35}$$

We numerically integrate the final step; we discretize $s_{inv,i}$ into a grid of 10^5 points, distributed equally between $\hat{s}_{inv,i} \pm 5\hat{\sigma}_{inv,i}$.

Model 3 Similar to model 2, we now include $s_{high,i}$ as a covariate,

bioRxiv preprint doi: https://doi.org/10.1101/2025.08.18.670924; this version posted August 21, 2025. The copyright holder for this preprint (which was not certified by peer review) is the author/funder, who has granted bioRxiv a license to display the preprint in perpetuity. It is made available under aCC-BY 4.0 International license.

$$w_i = a + b_{high} s_{high,i} + c_i + \epsilon_i \ , \tag{S36}$$

$$s_{high,i} \sim t(\hat{s}_{high,i}, \hat{\sigma}^2_{high,i}, n_i - 1) \ . \tag{S37}$$

The likelihood of model 3 is analogous to that of model 2.

Evaluating model performance We obtained parameter estimates for all three models by maximizing their respective likelihoods using `scipy.optimize.minimize`. We partition the variance in w_i across measurements, $\mathrm{var}(w_i)$, into a maximum of three components: variance explained by the invasion or high-frequency fitness effects (σ^2_s), measurement error (σ^2_m), and unexplained variance (σ^2_d). The estimate $\hat{\sigma}^2_d$ is directly obtained from the maximum likelihood estimate. We estimate $\hat{\sigma}^2_s = \mathrm{var}(\hat{a}+\hat{b}s_i)$, for each the invasion or high-frequency fitness effects. Then the variance associated with total measurement error is calculated as the remaining variance, $\hat{\sigma}^2_m = \mathrm{var}(w_i) - \hat{\sigma}^2_d - \hat{\sigma}^2_s$. Results are summarized in Table S2.

Model	$\hat{a}$	$\hat{b}$	$\hat{\sigma}^2_d$	$\hat{\sigma}^2_m$	$\hat{\sigma}^2_s$	$\log \hat{L}$	AIC
1	-0.12		0.012	0.0076		35.8	-67.6
2	-0.029	-0.26	0.0062	0.008	0.00554	43.4	-80.9
3	-0.11	-0.057	0.012	0.0061	0.0021	35.2	-64.4

Table S2: **Results from ANCOVA models.** Parameters fit via maximum likelihood. We find that Model 2 is the best fit to our data, both from its low Aikake Information Criteria (AIC), and from pairwise likelihood ratio tests ($p < 10^{-4}$).

S3 Within-cycle time-courses

S3.1 Coculture experiments

Previous competition experiments focused on solely measuring two time points, both at the end of a growth cycle, separated by one growth cycle. We reasoned that measuring the within-cycle dynamics may help us understand the frequency-dependent ecological dynamics of strain competition.

After the initial growth cycles of fluorescently tagged strains (as previously described in section S1.1), we combined the strains into two cocultures, setting the volumetric fraction of one strain to either 10% or 90%. These cocultures were grown for one additional 24-hour cycle in DM25 medium, after which we performed a flow cytometry measurement—this served as the baseline, or time 0. Immediately afterward, we prepared replicate cultures by diluting the overnight culture 1:100 into fresh DM25, vortexing thoroughly, and distributing 1mL aliquots into individual wells of a glass 96-well plate. Each condition was performed in triplicate (three biological replicates). The plate was incubated in a 37°C warm room, shaking at 180rpm. At regular intervals (approximately every 45 minutes) over a 10-hour period, we briefly removed the plate to collect 60µL subsamples for flow cytometry analysis, capturing the exponential phase and the onset of stationary phase. An additional sample was collected at 24 hours to mark the end of the growth cycle. Subsamples were discarded after measurement, and all sampling times were recorded.

To understand the time-dependent growth dynamics, we sought to compute both the "instantaneous" growth rates and fitness effects of all competitions. We defined the growth rate (in units of per hour) at time t as simply $\log(n_{t+\Delta t}) - \log(n_t)$, where Δt is the interval of time between measurements, and n_t is the total number of observed cells, after accounting for the measurement dilution factor. Analogously, we defined the fitness effect as $\mathrm{logit}(f_{t+\Delta t}) - \mathrm{logit}(f_t)$, where f_t is the frequency of the mutant relative to REL606. As computing derivatives is often quite noisy, we averaged over n_t and f_t for each time point t for all three biological replicates per condition. We computed the time-dependent growth rates and fitness effects via

bioRxiv preprint doi: https://doi.org/10.1101/2025.08.18.670924; this version posted August 21, 2025. The copyright holder for this preprint (which was not certified by peer review) is the author/funder, who has granted bioRxiv a license to display the preprint in perpetuity. It is made available under aCC-BY 4.0 International license.

`np.gradient`, and then applied a simple convolution/moving average to further reduce noise associated with computing derivatives–for an estimate x_t, we give the updated estimate as $0.5x_t + 0.25x_{t-1} + 0.25x_{t+1}$; the left boundary is given by $0.75x_t + 0.25x_{t+1}$, with an analogous equation for the right boundary. We then used standard bootstrapping–resampling with replacement–to compute the sampling errors associated with our estimates of growth rates and fitness effects.

S3.2 Measuring growth curves via plate reader

We sought to measure growth curves of all strain in monoculture. We prepared cultures as previously described, with six biological replicates per strain. To measure growth curves, we prepared 100µL cultures in DM25 in a flat bottom 96 well plate, and measured OD600 absorbance in a shaking plate reader (SpectraMax 190; Molecular Devices) over the course of 20 hours. We extracted an estimate of the average growth rate, r, by fitting an exponential curve to the portion of the growth curve in exponential growth, via standard ordinary least squares. We obtained an estimate by fitting the following model to the portions of the growth curves between the end of exponential phase and the beginning of stationary phase (5-10 hours),

$$\hat{y} \approx \begin{cases} y_0 e^{rt} & \text{for } t < T \\ y_0 e^{rT} & \text{for } t \geq T \end{cases} \tag{S38}$$

Here y is OD600 and t is time. We fit the parameters y_0, r, and T simultaneously using ordinary least squares. Results are shown in Figure S14.

S4 Batch culture resource competition model

As stated in the main text, we consider a simple model that tracks the dynamics of strain abundance, $n(t)$, and resource abundance, $R(t)$,

$$\dot{n}_{wt}(t) = r_{wt}\, b(R(t)) n_{wt}(t) \ , \tag{S39}$$
$$\dot{n}_{mut}(t) = r_{mut}\, b(R(t)) n_{mut}(t) \ , \tag{S40}$$
$$\dot{R}(t) = -r_{wt} a_{wt} n_{wt}(t) b(R(t)) - r_{mut} a_{mut} n_{mut}(t) b(R(t)) \ . \tag{S41}$$

Analytical calculations We will first choose $b(R(t))$ to be a simple step function $\theta(R(t))$ for our analytical calculations. We also simulate the dynamics under other choices of $b(R(t))$ (Figure S11-S12). We are able to analyze the dynamics analytically in two limits, when $n_{wt}(t) \gg n_{mut}(t)$ and $n_{wt}(t) \ll n_{mut}(t)$. We call whichever strain that is in the majority, maj; in the limit that $f_{maj} \to 1$, the resource dynamics reduce to,

$$\dot{R}(t) = -r_{maj} a_{maj} n_{maj}(t) \ . \tag{S42}$$

Integrating yields,

$$R(t) = R_0 + a_{maj} n_{maj}(0) \left(1 - e^{r_{maj} t}\right) \ . \tag{S43}$$

We can then find T where $R(T) = 0$,

$$T = \frac{1}{r_{maj}} \log\left(\frac{R_0}{a_{maj} n_{maj}(0)} + 1\right) \ . \tag{S44}$$

Fitness effects are similarly easy to calculate,

$$s = \Delta \log n_{mut} - \Delta \log n_{wt} \tag{S45}$$
$$= \log n_{mut}(0) e^{r_{mut} T} - \log n_{mut}(0) - \log n_{wt}(0) e^{r_{wt} T} + \log n_{wt}(0) \tag{S46}$$
$$= T(r_{mut} - r_{wt}) \ . \tag{S47}$$

bioRxiv preprint doi: https://doi.org/10.1101/2025.08.18.670924; this version posted August 21, 2025. The copyright holder for this preprint (which was not certified by peer review) is the author/funder, who has granted bioRxiv a license to display the preprint in perpetuity. It is made available under aCC-BY 4.0 International license.

We can in fact obtain exact solutions to the ODEs for many monotonically increasing $b(R)$, again in the limits $n_{wt}(t) \gg n_{mut}(t)$ or $n_{wt}(t) \ll n_{mut}(t)$. To see that, we note that in those limits,

$$\dot{n}_{maj}(t) = r_{maj}\, b(R(t)) n_{maj}(t) = -\frac{\dot{R}(t)}{a_{maj}} \ , \tag{S48}$$

$$n_{maj}(t) = n_0 + \frac{R_0 - R(t)}{a_{maj}} \ . \tag{S49}$$

So, the system can be reduced to a single ODE,

$$\frac{1}{r_{maj}} \dot{R}(t) = b(R) \left[R - a_{maj} n_0 - R_0 \right] \ . \tag{S50}$$

This equation can be exactly solved for $b(R) = R^n$ for all positive integers n, for Monod's equation $b(R) = \frac{R}{k+R}$, and various other choices (e.g. $b(R) = \sqrt{R}$, $b(R) = \frac{R^n}{k+R^n}$, etc). This equation makes it clear that the timescale of resource depletion will always depend inversely on the growth rate of the strain in the majority, $T \propto r_{maj}^{-1}$, because we will always be able to rescale time by r_{maj}.

In the case that $b(R) = R$ (the choice of MacArthur's consumer-resource model), the population abundance dynamics follow a logistic equation,

$$n(t) = \frac{n_0 \left(a_{maj} n_0 + R_0 \right)}{a_{maj} n_0 + R_0 e^{-r_{maj} t (a_{maj} n_0 + R_0)}} \ . \tag{S51}$$

Non-transitivity Our model predicts that fitness effects will generally not be transitive across environments. We can calculate the invasion and high-frequency non-transitivities under our resource competition model (equations S7-S10),

$$\nu_{inv} \propto \left(\frac{1}{r_3} - \frac{1}{r_2} \right) (r_1 - r_3) \ , \tag{S52}$$

$$\nu_{high} \propto \left(\frac{1}{r_3} - \frac{1}{r_1} \right) (r_3 - r_2) \ . \tag{S53}$$

It is straightforward to calculate the expected non-transitivity under the other definitions of non-transitivity (equations S11-S18). As we see, the magnitude of non-transitivity generally increases as the difference between specific pairs of growth rates increases. Invasion and high-frequency non-transitivity will generally be non-zero unless either $r_3 = r_2$ or $r_1 = r_3$.

Frequency-dependent slope We can compute the expected relationship between the invasion fitness of a mutant, s_{inv}, and the frequency-dependent slope (Figure S13). Here, we simply take the frequency-dependent slope to be $\Delta s = s_{high} - s_{inv}$. This differs from the frequency-dependent slope calculated from our data, w–while w was computed as the slope of a fitted linear regression to the data, Δs is computed by taking the slope between the fitness effects as the frequency approaches zero or one. If the form of $s(f)$ is actually linear, then the two slopes should be equivalent to each other, $w = \Delta s$. Deviations from linearity will, in general, cause $w \neq \Delta s$; however, the difference in w and Δs will presumably be small, assuming that $s(f)$ is not ill-behaved.

For generality, we lump the prefactor of T into a new constant, c, so that $T = c/r_{maj}$. Following some algebra, we calculate Δs as,

$$\Delta s = -s_{inv} \left(\frac{1}{\frac{c}{s_{inv}} + 1} \right) \ . \tag{S54}$$

bioRxiv preprint doi: https://doi.org/10.1101/2025.08.18.670924; this version posted August 21, 2025. The copyright holder for this preprint (which was not certified by peer review) is the author/funder, who has granted bioRxiv a license to display the preprint in perpetuity. It is made available under aCC-BY 4.0 International license.

This expression can be further simplified in two limits. When s_{inv} is large and positive, $c \ll s_{inv}$, the slope will be linearly related to the invasion fitness,

$$\Delta s = -s_{inv} \; . \tag{S55}$$

In the opposite limit, $c \gg s_{inv}$,

$$\Delta s = -\frac{s_{inv}^2}{c} \; . \tag{S56}$$

References

[1] Joao A. Ascensao, Jonas Denk, Kristen Lok, QinQin Yu, Kelly M. Wetmore, and Oskar Hallatschek. Rediversification following ecotype isolation reveals hidden adaptive potential. *Current Biology*, 34(4):855–867.e6, February 2024.

[2] Rudolf O. Schlechter, Hyunwoo Jun, Michał Bernach, Simisola Oso, Erica Boyd, Dian A. Muñoz-Lintz, Renwick C.J. Dobson, Daniela M. Remus, and Mitja N.P. Remus-Emsermann. Chromatic Bacteria - A Broad Host-Range Plasmid and Chromosomal Insertion Toolbox for Fluorescent Protein Expression in Bacteria. *Frontiers in microbiology*, 9(DEC), 12 2018.

bioRxiv preprint doi: https://doi.org/10.1101/2025.08.18.670924; this version posted August 21, 2025. The copyright holder for this preprint (which was not certified by peer review) is the author/funder, who has granted bioRxiv a license to display the preprint in perpetuity. It is made available under aCC-BY 4.0 International license.

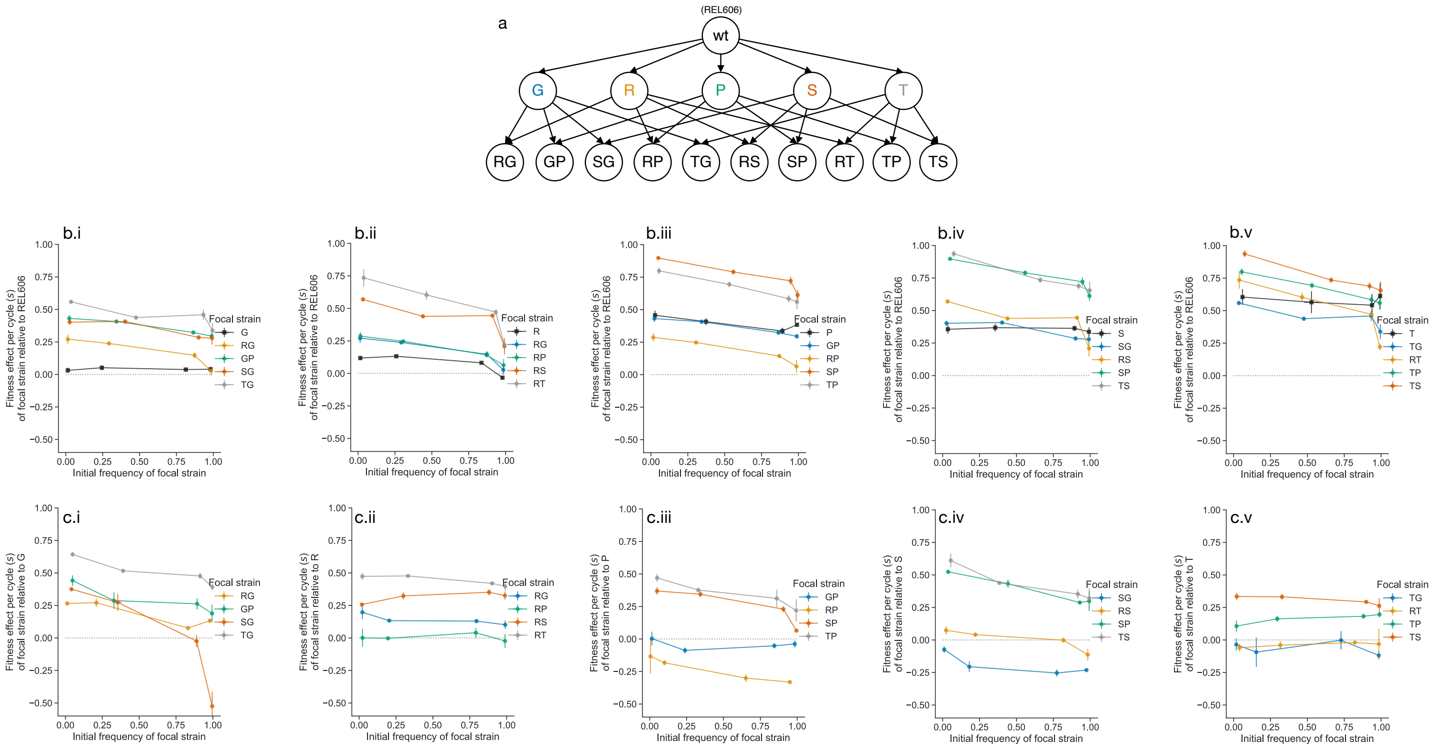

Figure S1: **Ubiquity of frequency-dependence.** Identical to Figure 1; linear x-axis instead of a logit x-axis. (**a**) We study a set of single and double mutants, all derived from the ancestor of the *E. coli* LTEE, REL606. (**b**) We measured the fitness effects, s, of all single and double mutants against REL606 as a function of frequency. Each subpanel (i-v) shows the measured frequency-dependent fitness effects for each single mutant and derived double mutants. Note that we plot the frequency-dependent fitness effects of double mutants twice–once on each subpanel corresponding to an immediate ancestral single mutant–to allow for comparison. (**c**) We additionally measured the frequency-dependent fitness effects of all double mutants against their single mutant ancestors. Error bars represent standard errors across biological replicates (n =4~6).

bioRxiv preprint doi: https://doi.org/10.1101/2025.08.18.670924; this version posted August 21, 2025. The copyright holder for this preprint (which was not certified by peer review) is the author/funder, who has granted bioRxiv a license to display the preprint in perpetuity. It is made available under aCC-BY 4.0 International license.

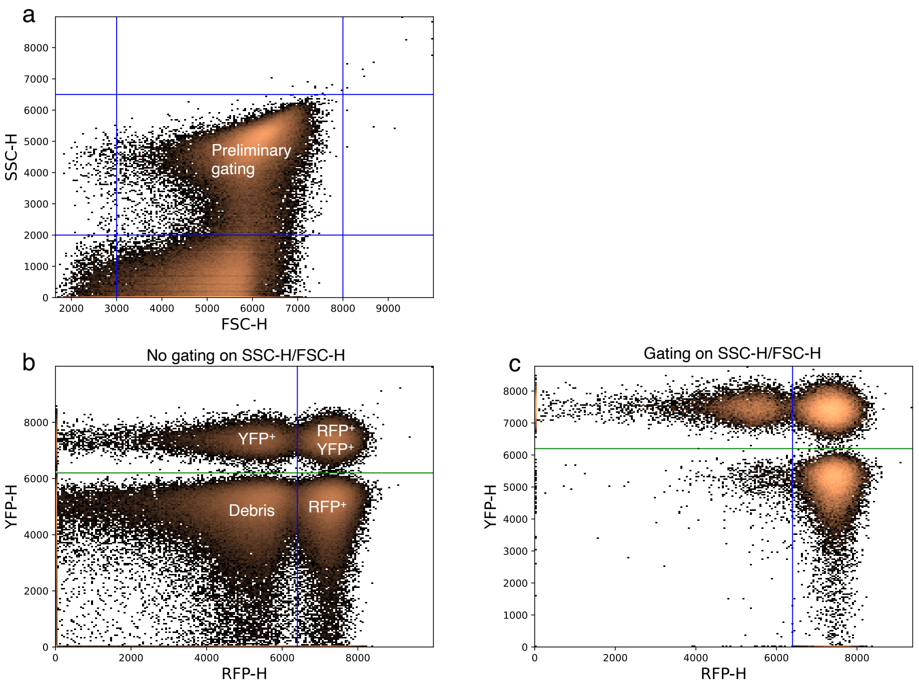

Figure S2: **Example of flow cytometry gating strategy.** (a) We first gate on FSC-H/SSC-H to select for the cells present in our sample, and deplete debris. (b) In the absence of the initial FSC-H/SSC-H, we see a large cloud of debris particles in the bottom left corner, which (c) largely disappears after the initial gating. We use threshold gates to divide the population into YFP^+, RFP^+, and double positive events. The double positive events correspond to coincident events (which are difficult to eliminate due to the small size of *E. coli*). We use a previously validated pipeline [1] to estimate the frequency of each fluorescently labeled subpopulation, accounting for coincident events.

bioRxiv preprint doi: https://doi.org/10.1101/2025.08.18.670924; this version posted August 21, 2025. The copyright holder for this preprint (which was not certified by peer review) is the author/funder, who has granted bioRxiv a license to display the preprint in perpetuity. It is made available under aCC-BY 4.0 International license.

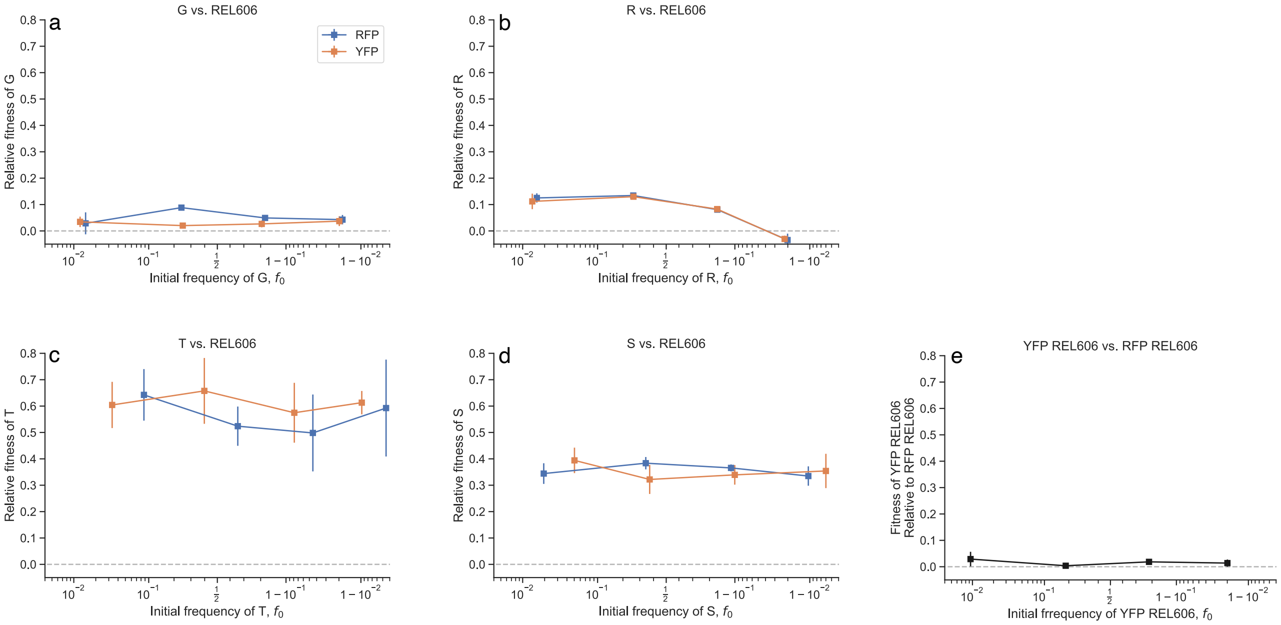

Figure S3: **Fluorophores do not differentially impact fitness.** (a-d) We conducted several control experiments where we competed a either an RFP-tagged mutant against a YFP-tagged REL606 clone, or vice-versa. We measured the fitness effects, s, of all mutants against REL606 as a function of frequency. Frequency-dependent fitness effects after switching fluorophores are generally within error bars. (e) Similarly, we competed a YFP-tagged REL606 clone against an RFP-tagged REL606 clone; fitness effects across all measured frequencies are not significantly different from zero. Error bars represent standard errors.

bioRxiv preprint doi: https://doi.org/10.1101/2025.08.18.670924; this version posted August 21, 2025. The copyright holder for this preprint (which was not certified by peer review) is the author/funder, who has granted bioRxiv a license to display the preprint in perpetuity. It is made available under aCC-BY 4.0 International license.

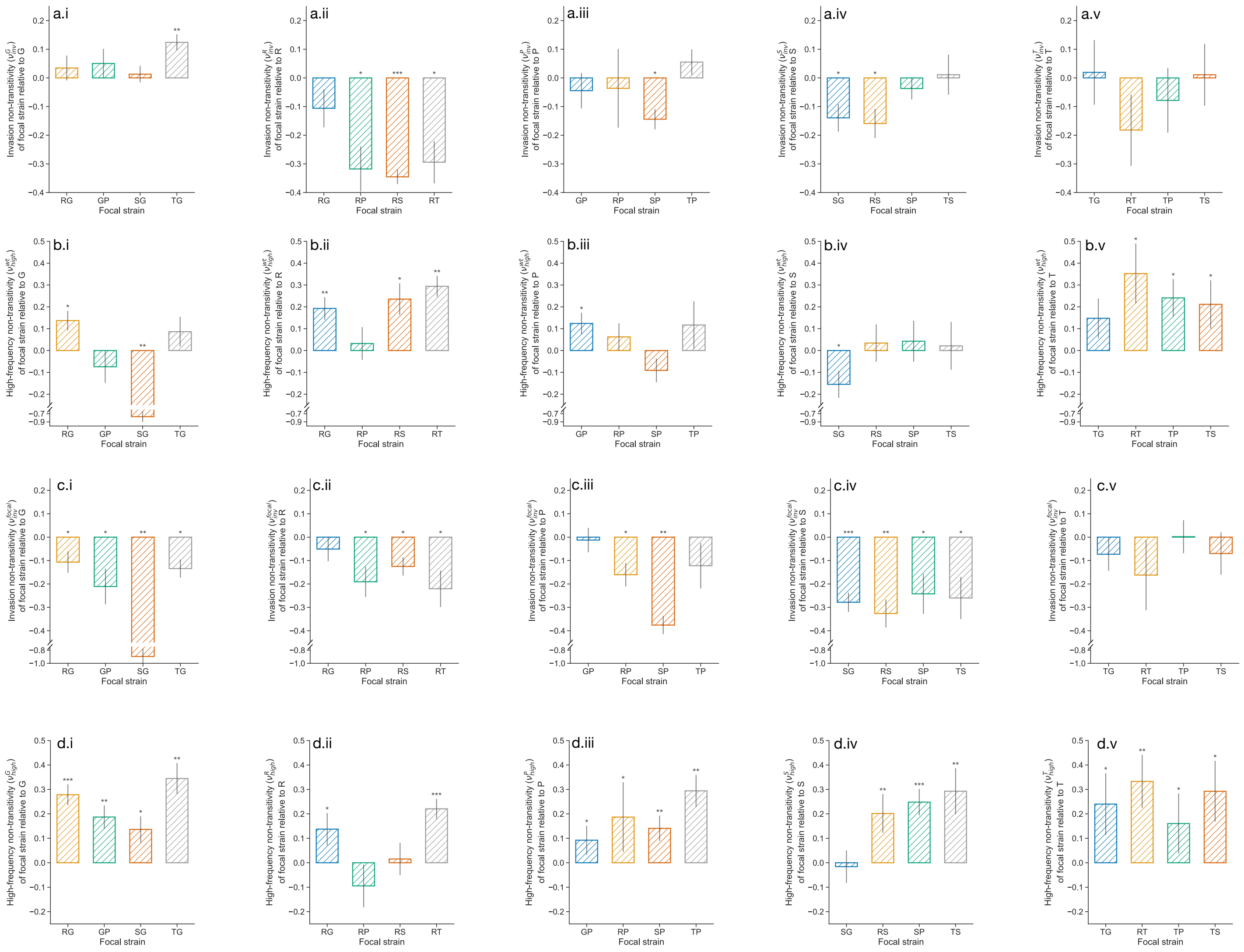

Figure S4: **Non-transitivity of fitness effects (related to Figure 2).** Quantification of non-transitivity using alternative definitions of frequency-dependent non-transitivity (equations S11-S18). Each row (a-d) represents a different definition of non-transitivity. Each column (i-v) represents non-transitivity of double mutants against different single mutants, (i) G, (ii) R, (iii) P, (iv) S, (v) T. Note that the y axis scales differ between rows. * $p < 0.05$, ** $p < 0.01$, *** $p < 0.001$, post-FDR correction.

bioRxiv preprint doi: https://doi.org/10.1101/2025.08.18.670924; this version posted August 21, 2025. The copyright holder for this preprint (which was not certified by peer review) is the author/funder, who has granted bioRxiv a license to display the preprint in perpetuity. It is made available under aCC-BY 4.0 International license.

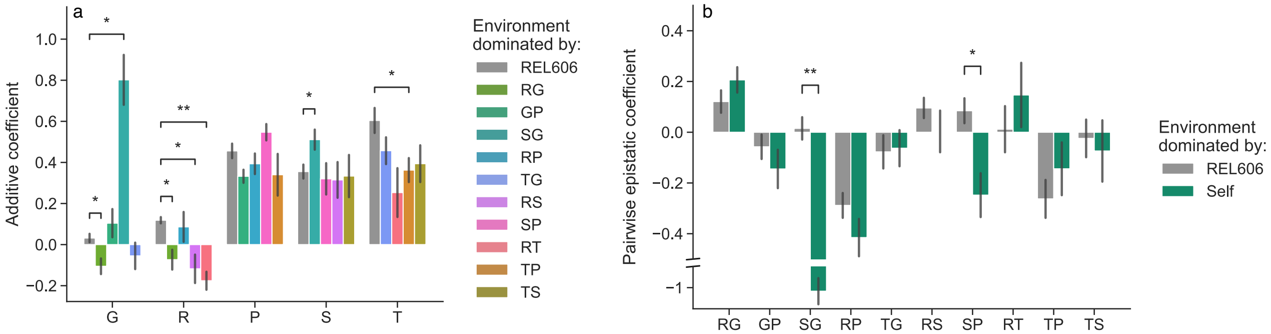

Figure S5: **Frequency-dependent epistasis.** Estimation of (a) additive and (b) epistatic coefficients of double and single mutants. Coefficients are calculated using fitness effects at either invasion or high frequencies; we compare coefficients when different clonal strains are in the vast majority of the population, and thus dominating the environment. Coefficients are calculated using the methods presented in section S2.3. * $p < 0.05$, ** $p < 0.01$, *** $p < 0.001$, post-FDR correction.

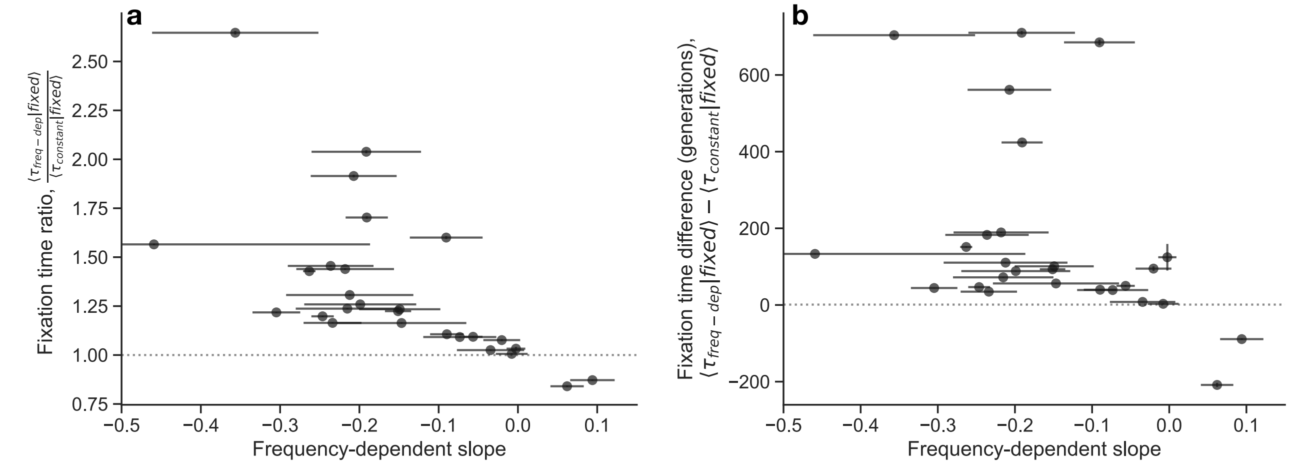

Figure S6: **Comparison of simulated mutant fixation time estimates with and without frequency-dependent fitness effects**. We numerically simulated the frequency (f) dynamics of every strain pair in our dataset by numerically integrating the one-locus Langevin equation, $\frac{df}{dt} = s(f)f(1-f) + \sqrt{\frac{f(1-f)}{N_e}}\eta(t)$, where $\eta(t)$ is standard Gaussian white noise, and we set $N_e = 10^6$. We either approximate $s(f)$ as a linear function with previously estimated parameters (see Figure 2 in the main text), or we set $s(f)$ to be constant (at the invasion fitness of the linear approximation). We simulate the frequency dynamics for each parameter set (corresponding to each strain competition) 2000 times, with the focal strain set to an initial frequency of 10^{-6}; if the focal strain fixed, we recorded the time to fixation. We compare the time to fixation with frequency-dependent or constant fitness effects by either taking (a) the ratio of average fixation times or (b) the difference in average fixation times.

bioRxiv preprint doi: https://doi.org/10.1101/2025.08.18.670924; this version posted August 21, 2025. The copyright holder for this preprint (which was not certified by peer review) is the author/funder, who has granted bioRxiv a license to display the preprint in perpetuity. It is made available under aCC-BY 4.0 International license.

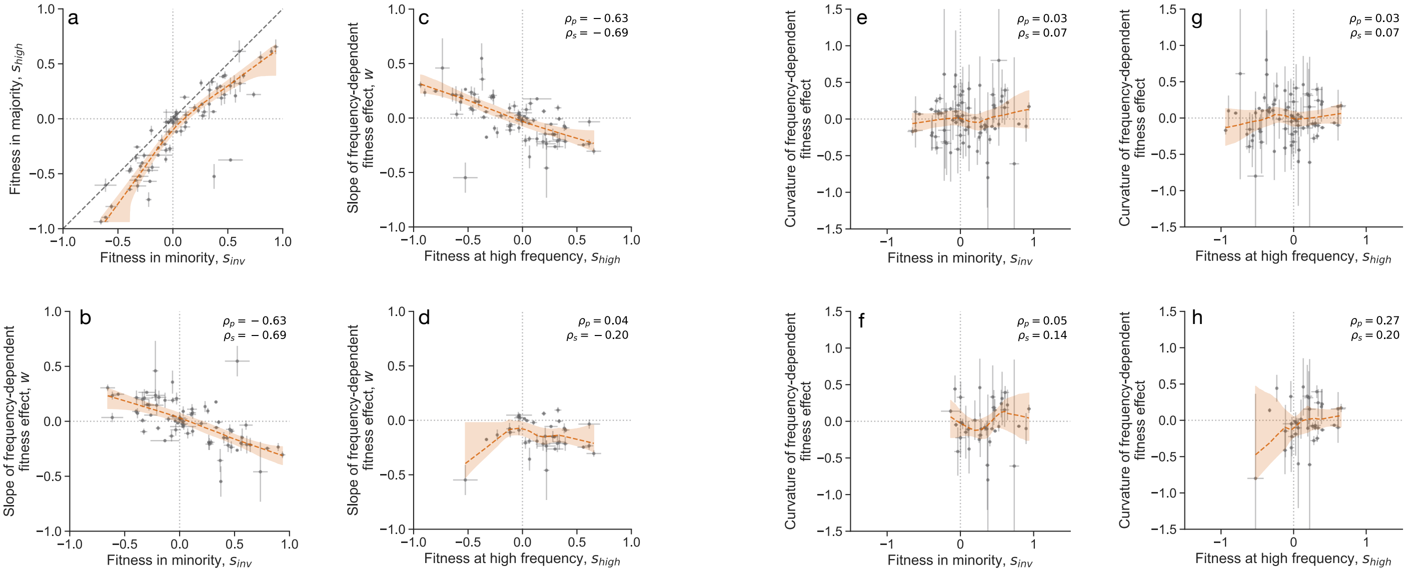

Figure S7: **Statistical relationships between invasion/high-frequency fitness effects and frequency dependence (related to Figure 4).** Specifically, predictors of (a-d) the average slope of the fitness effects, and (e-h) the curvature of fitness effects. (a-b) Analogous to Figure 4a-b, but we treat each experiment as two points, where either strain in the competition can be the focal or competitor strain. (c) Relationship between the fitness at high frequency and frequency-dependent slope; (d) analogous, but only considering points where the competitor strain is more ancestral relative to the focal strain. Relationship between curvature of frequency-dependence and (e-f) invasion fitness effect or (g-h) fitness effect at high frequency; either considering (e,g) points from both perspectives, or (f,h) only points where the competitor strain is more ancestral relative to the focal strain. Red lines represent LOESS regression fit; shaded region is a 95% confidence interval (obtained from bootstrapping). Error bars on points represent standard errors. Here, ρ_p is the estimated Pearson's correlation coefficient; ρ_s is the estimated Spearman's correlation coefficient.

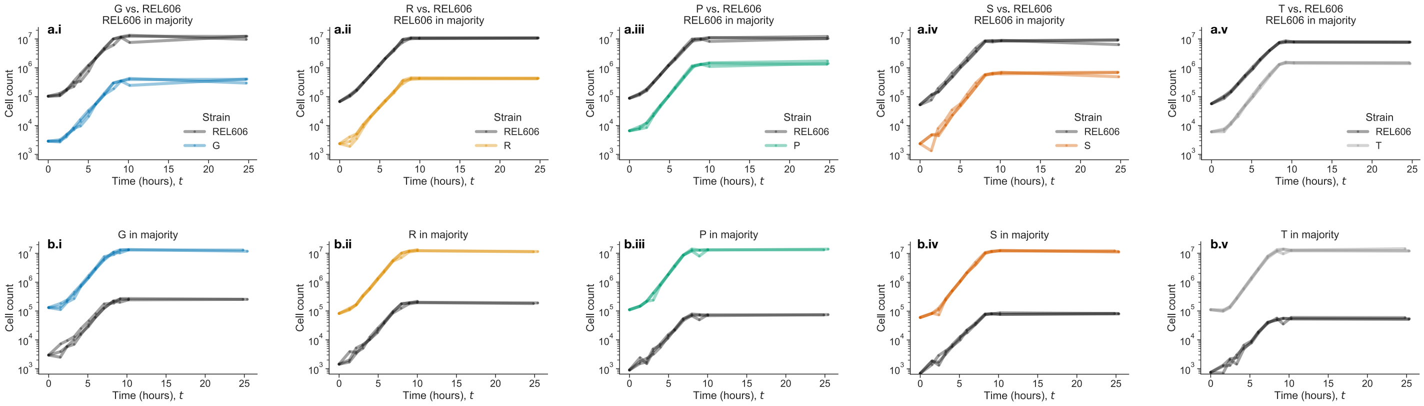

Figure S8: **Within-cycle population dynamics, including the 24 hour time point (related to Figure 5).** (a-b) Dynamics of cell counts over the course of the entire growth cycle; where either (a) REL606 is in the majority of the population, or (b) the mutant is in the majority of the population. Trajectories are shown for all three biological replicates per condition. Population sizes are approximately constant from 10-24 hours, as expected.

bioRxiv preprint doi: https://doi.org/10.1101/2025.08.18.670924; this version posted August 21, 2025. The copyright holder for this preprint (which was not certified by peer review) is the author/funder, who has granted bioRxiv a license to display the preprint in perpetuity. It is made available under aCC-BY 4.0 International license.

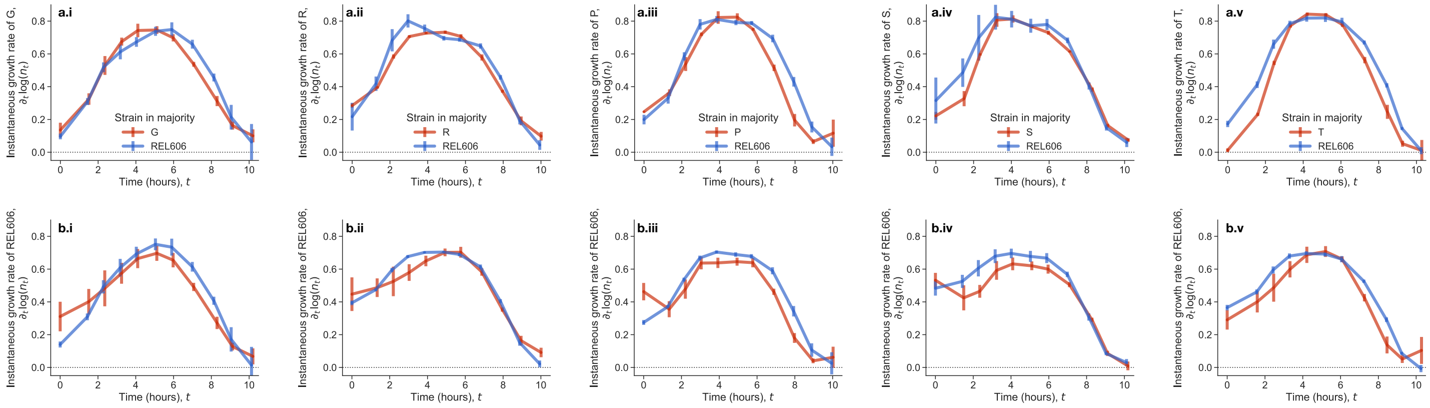

Figure S9: **Within-cycle population growth rates (related to Figure 5).** Estimates of the time-dependent growth rates of (a) the mutants and (b) REL606 in each experiment. Error bars represent standard errors.

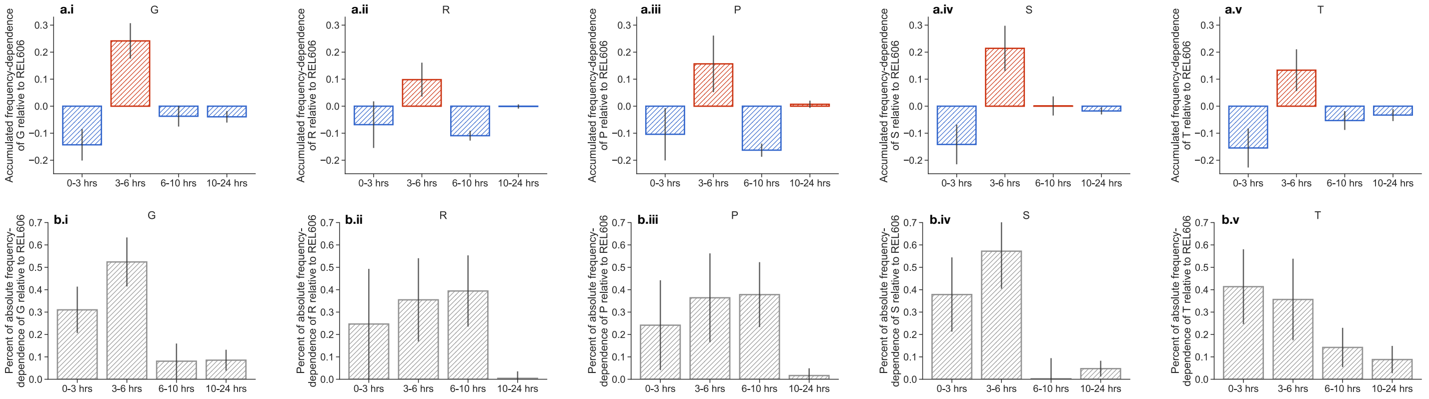

Figure S10: **Accumulated frequency-dependence over time intervals (related to Figure 5d).** (a) We subtracted the total accumulated fitness effects at each time interval to get an estimate of the time-dependence frequency-dependence, $s_{high} - s_{inv}$ (fitness at high mutant frequency - low mutant frequency). (b) We computed the percent contribution of each time bin to the total absolute frequency-dependence, i.e. $|s_{i,high} - s_{i,inv}| / \sum_j |s_{j,high} - s_{j,inv}|$. Error bars represent standard errors.

bioRxiv preprint doi: https://doi.org/10.1101/2025.08.18.670924; this version posted August 21, 2025. The copyright holder for this preprint (which was not certified by peer review) is the author/funder, who has granted bioRxiv a license to display the preprint in perpetuity. It is made available under aCC-BY 4.0 International license.

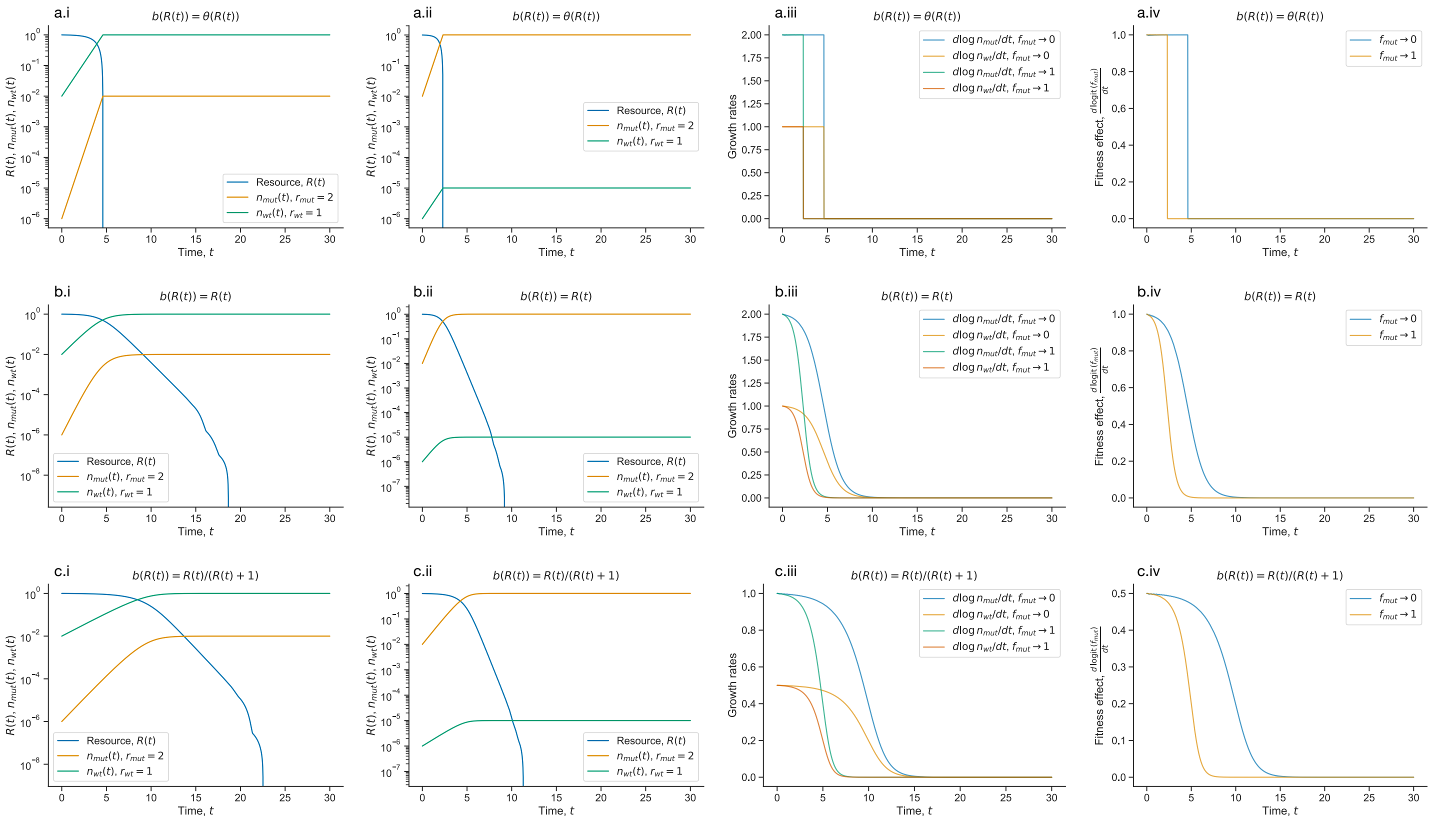

Figure S11: **Examples of simulations of batch culture resource competition dynamics.** Here we show examples of simulated batch culture resource competition dynamics, obtained by numerically solving equations 2-4 (in the main text). We choose three different values of $b(R(t))$, (a) a step function, (b) a linear function, and (c) a Monod function. We varied the initial frequency of the mutant strain, using either (i) $f = 10^{-4}$ or (ii) $f = 1 - 10^{-4}$. We also compute and compare (iii) population growth rates, along with (iv) instantaneous fitness effects. Parameter values: initial amount of resource, $R_0 = 1$; initial total population size $N = 10^{-2}$; $a_{wt} = a_{mut} = 1$.

bioRxiv preprint doi: https://doi.org/10.1101/2025.08.18.670924; this version posted August 21, 2025. The copyright holder for this preprint (which was not certified by peer review) is the author/funder, who has granted bioRxiv a license to display the preprint in perpetuity. It is made available under aCC-BY 4.0 International license.

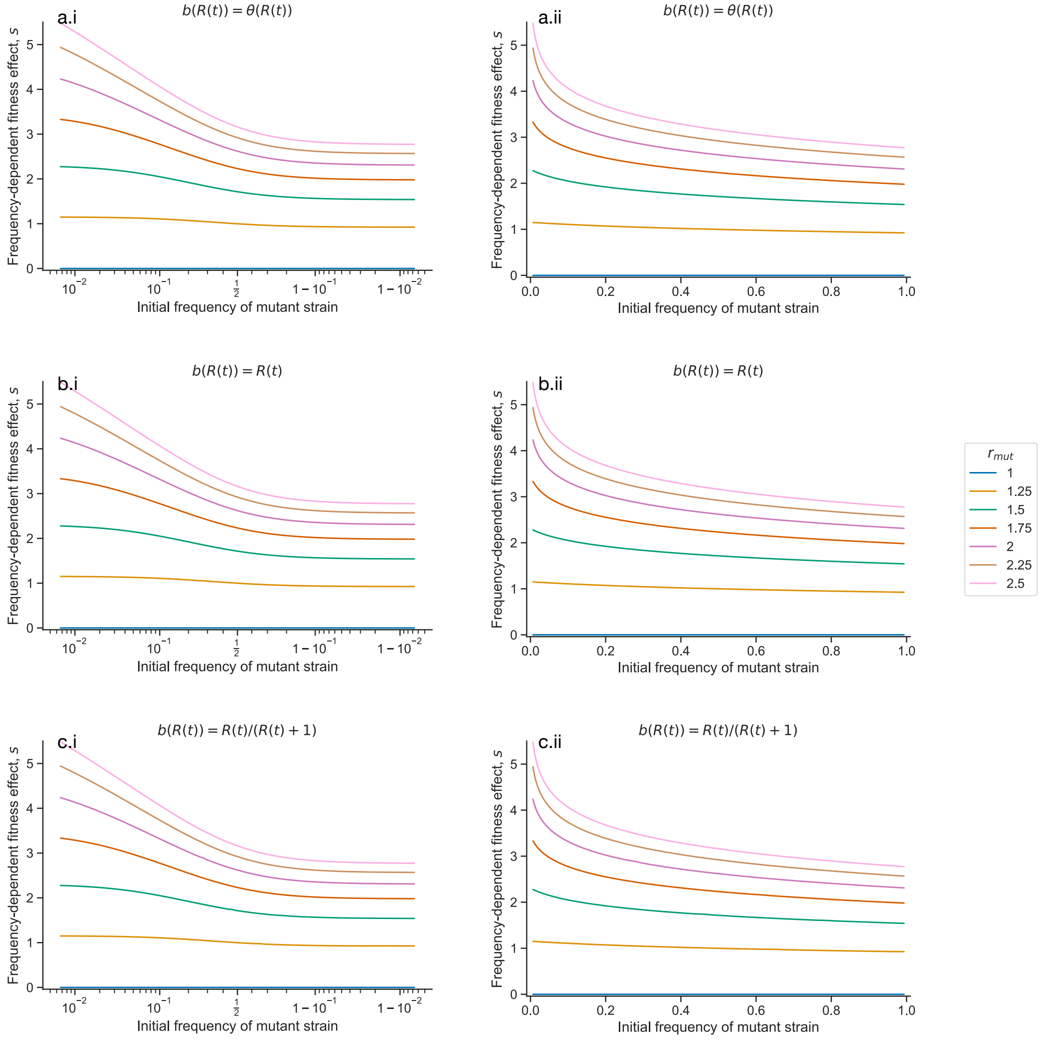

Figure S12: **Frequency-dependence arising from resource competition dynamics.** Here we show examples of the effective negative frequency-dependent fitness effects that arise from batch culture resource competition dynamics, obtained by numerically solving equations 2-4 (in the main text). We choose three different values of $b(R(t))$, (a) a step function, (b) a linear function, and (c) a hill function. We show the same plots on either a (i) logit-scaled x-axis, or a (ii) linear-scaled x-axis. Parameter values: wild-type growth rate, $r_{wt} = 1$, initial amount of resource, $R_0 = 1$; initial total population size $N = 10^{-2}$; $a_{wt} = a_{mut} = 1$.

bioRxiv preprint doi: https://doi.org/10.1101/2025.08.18.670924; this version posted August 21, 2025. The copyright holder for this preprint (which was not certified by peer review) is the author/funder, who has granted bioRxiv a license to display the preprint in perpetuity. It is made available under aCC-BY 4.0 International license.

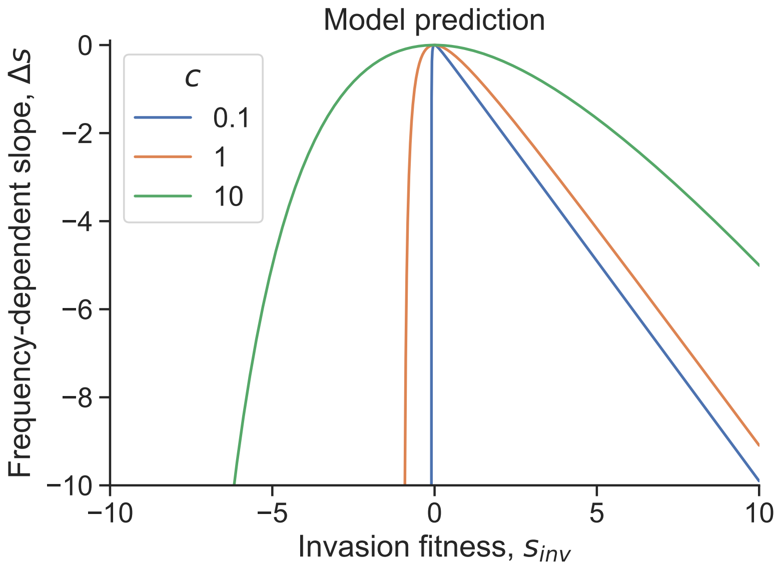

Figure S13: **Frequency-dependent slopes predicted from resource competition dynamics.** Prediction for the frequency-dependent slope from equation S54, varying c. We held $r_{wt} = 1$ constant and $a_{wt} = a_{mut} = 1$, and varied r_{mut} to change s_{inv}.

bioRxiv preprint doi: https://doi.org/10.1101/2025.08.18.670924; this version posted August 21, 2025. The copyright holder for this preprint (which was not certified by peer review) is the author/funder, who has granted bioRxiv a license to display the preprint in perpetuity. It is made available under aCC-BY 4.0 International license.

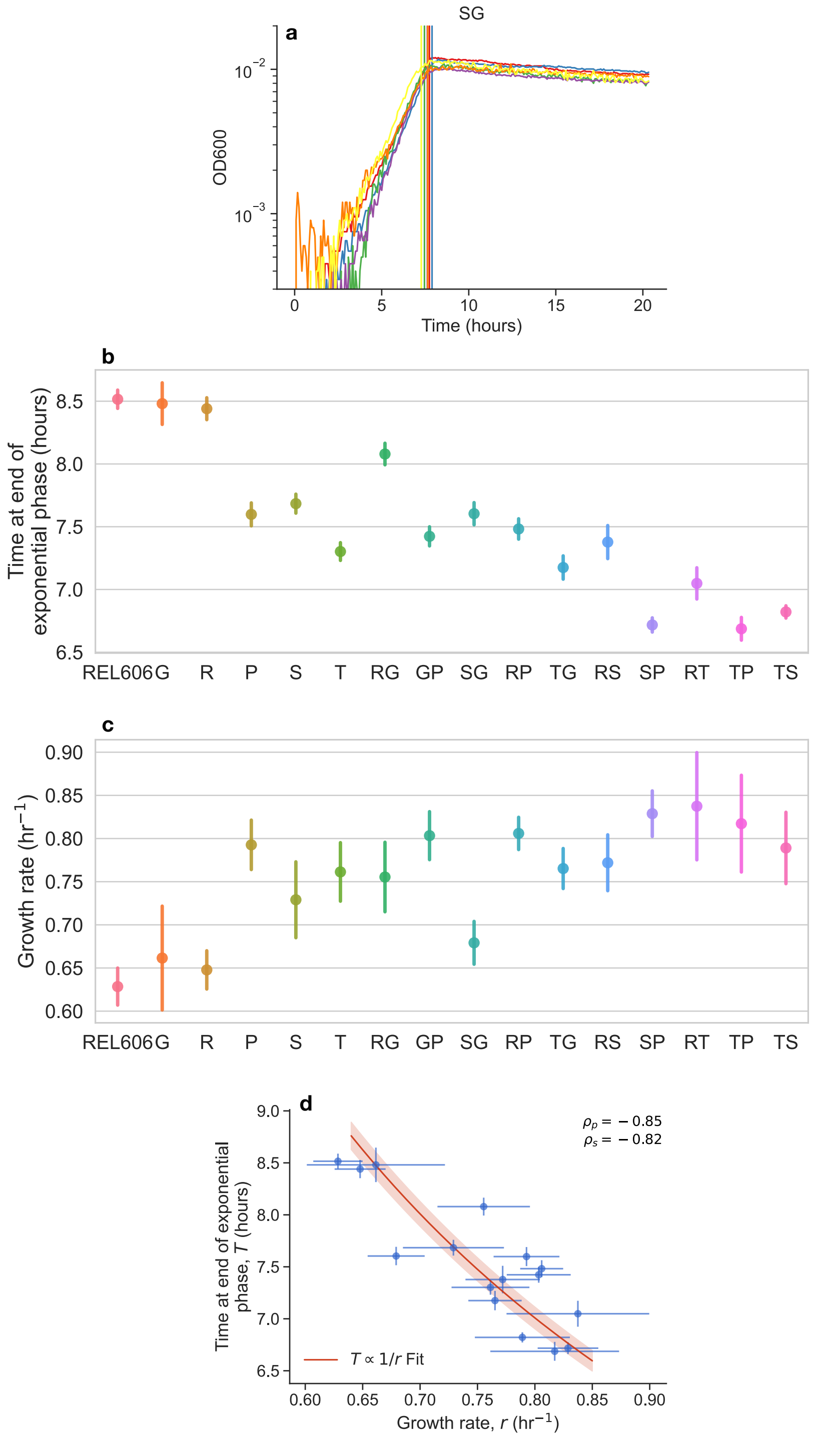

Figure S14: **Growth curves of monocultures from plate reader.** We measured growth curves using a plate reader of all 16 strains (six biological replicates per strain); (a) example of growth curves, along with estimated time at the end of exponential phase. We estimated (b) the time at the end of exponential phase (T) and (c) average growth rate (r) for each strain. (d) We see a strong negative correlation between r and and T, consistent with a $T \propto 1/r$ relationship. All error bars represent standard errors. ρ_p represents Pearson's correlation; ρ_s represents Spearman's correlation.
